# *Ostreopsis* Schmidt and *Coolia* Meunier (Dinophyceae, Gonyaulacales) from Cook Islands and Niue (South Pacific Ocean), including description of *Ostreopsis tairoto* sp. nov.

**DOI:** 10.1038/s41598-023-29969-z

**Published:** 2023-02-22

**Authors:** A. Verma, M. Hoppenrath, K. F. Smith, J. S. Murray, D. T. Harwood, J. M. Hosking, T. Rongo, L. L. Rhodes, S. A. Murray

**Affiliations:** 1 School of Life Sciences, University of Technology, Broadway, Sydney, NSW 2007 Australia; 2 Senckenberg am Meer, German Center for Marine Biodiversity Research (DZMB), Südstrand 44, D-26382 Wilhelmshaven, Germany; 3 Cawthron Institute, Nelson, 7010 New Zealand; 4 Te Ipukarea Society, PO Box 649, Rarotonga, Cook Islands; 5 Kōrero O Te `Ōrau, Avarua, PO Box 881, Avarua, Rarotonga, Cook Islands

**Keywords:** Ecology, Microbiology, Molecular biology, Ecology

## Abstract

It is important to decipher the diversity and distribution of benthic dinoflagellates, as there are many morphologically indistinct taxa that differ from one another in production of potent toxins. To date, the genus *Ostreopsis* comprises twelve described species, of which seven are potentially toxic and produce compounds presenting a threat to human and environmental health. In this study, isolates previously identified as “*Ostreopsis* sp. 3” were sampled from the area where it was first reported, Rarotonga, Cook Islands, and have been taxonomically and phylogenetically characterised as *Ostreopsis tairoto* sp. nov. Phylogenetically, the species is closely related to “*Ostreopsis* sp. 8”, *O. mascarenensis*, “*O*. sp. 4”, *O. fattorussoi, O. rhodesiae* and *O*. cf. *siamensis*. Previously, it was considered a part of the *O*. cf. *ovata* complex but can be distinguished from *O*. cf. *ovata* based on the small pores identified on this study, and from *O. fattorussoi* and *O. rhodesiae* based on relative lengths of the 2′ plates. No known palytoxin -like compounds were detected in strains investigated in this study. Strains of *O. lenticularis, Coolia malayensis* and *C. tropicalis* were also identified and described. This study advances our knowledge of biogeography, distribution, and toxins of *Ostreopsis* and *Coolia* species.

## Introduction

Dinoflagellates have been studied extensively due to their potential to synthesize potent toxic molecules and forming harmful algal blooms (HABs), thereby impacting coastal resources and human health^1–3^. Efforts to understand the changing distributions of HAB species in response to ocean climate change are hampered by unclear identifications of taxa and the presence of cryptic and/or pseudo-cryptic diversity amongst several culprit genera^3–7^. The cosmopolitan HAB genus *Ostreopsis* Schmidt, occurs worldwide, but mainly in warm waters, and is associated with a variety of benthic/epiphytic habitats^8,9^. *Ostreopsis* was first described by Schmidt in 1901 from the Gulf of Thailand, with *O. siamensis* as the type species^10^. Traditionally, nine *Ostreopsis* species had been described based on morphology and thecal plate pattern, namely: *O. siamensis* Schmidt (1901), *O. lenticularis* Y.Fukuyo and *O. ovata* Y.Fukuyo (1981), *O. heptagona* D.R.Norris, J.W.Bomber & Balech (1985), *O. mascarenensis* J.P.Quod (1994) emend. Chomérat & J.-P.Quod (2020), *O. labens* M.A.Faust & S.L.Morton (1995), *O. belizeana* M.A.Faust, *O. caribbeana* M.A.Faust and *O. marina* M.A.Faust (1999). Only *O. fattorussoi* Accoroni, Romagnoli & Totti (2016), *O. rhodesiae* Verma, Hoppenrath & Murray (2016), and now the emended *O. mascarenensis, O. lenticularis* and *O. siamensis* have been described with both molecular and morphological information^11–19^.

*Ostreopsis* species are known to co-occur with other epi-benthic genera, such as *Coolia* Meunier that has a cosmopolitan distribution ranging from tropical to temperate waters^20–26^. *Coolia* species show homology with genus *Ostreopsis* in thecal plate tabulation, and at one time *C. monotis* Meunier (1919), the only species described until 1995, was proposed for transfer to *Ostreopsis* as *O. monotis* Lindemann (1928)^23,27^. It was later moved to *Glenodinium* Ehrenberg based on hypotheca tabulation^28^. A detailed morphological study conducted by Balech reinstated *Coolia* as a recognised genus^29^. Since then seven species have been described, namely: *C. tropicalis* M.A.Faust (1995), *C. areolata* Ten-Hage,Turquet, Quod & Couté (2000), *C. canariensis* S.Fraga (2008), *C. malayensis* Leaw, P.T.Lim & Usup (2010), *C. palmyrensis* Karafas, Tomas, York (2015), *C. santacroce* Karafas, Tomas, York (2015) and *C. guanchica* H.David, Laza-Martínez, F.Rodríguez & S.Fraga (2020)^20,26,30–34^.

The combination of genetic and morphometric tools has aided in the description of several new species from these genera, but the highly variable morphology of these species and lack of strains from type locality of certain species has also caused inaccurate or ambiguous species delimination^16,19^. In the case of *Ostreopsis*, the morphological variation derived from culture (non-natural) conditions increase the uncertainty. Tentative classifications such as *Ostreopsis* cf. *ovata* and *O.* cf. *siamensis* and undescribed ribotypes or clades (*Ostreopsis* sp. 1–9) emerged, the latter from phylogenetic studies^19,35–37^. Several such ‘ribotypes’ are morphologically indistinguishable and remain undescribed. Amongst *Coolia* spp., molecular data are available for all presently described species, except for *C. areolata*, but this species can be easily identified using morphological features^3,32^. Taxonomic identifications are important for species of these genera, as some *Ostreopsis* species are known to produce numerous palytoxin (PLTX) -like compounds and form HABs, which are known to cause human poisonings and the mortality of benthic invertebrates^38–45^. Also, four of the eight *Coolia* species, namely *C. tropicalis*, *C. malayensis*, *C. palmyrensis*, and *C. santacroce* are known to be toxic^33,46–49^.

One approach for resolving these taxonomic issues is to isolate strains from type localities, and to obtain extensive data from them including molecular genetic sequences (e.g. Tillmann et al.^50^). Chomérat et al.^18^ examined material from the type locality of *Ostreopsis lenticularis* and concluded that it was morphologically similar to the original description by Fukuyo^13^ and genetically similar to “*Ostreopsis* sp. 5”^35,37^, determining that “*Ostreopsis* sp. 5” is *O. lenticularis*. Phylogenetic and morphological studies of samples from Réunion Island, the type locality of *O. mascarenensis* by Chomérat et al.^16^ confirmed that *O. mascarenensis* forms a new lineage among *Ostreopsis* species, and it does not correspond to any of the unidentified ribotypes shown in previous studies. However, its relationship with *O. fattorussoi, O. rhodesiae,* and ribotypes “*Ostreopsis* sp. 3” and “*Ostreopsis* sp. 4” remained unclear. Chomérat et al.^51^ provided morphological and genetic support to separate the “temperate *O*. cf. *siamensis”* as a species distinct from “*Ostreopsis* sp. 6”. Later, Nguyen-Ngoc et al.^19^ concluded that “*Ostreopsis* sp. 6” is identical to the original description of *O. siamensis*, designated an epitype and presented an emended description of the species from Vietnamese waters.

**Figure 1:**
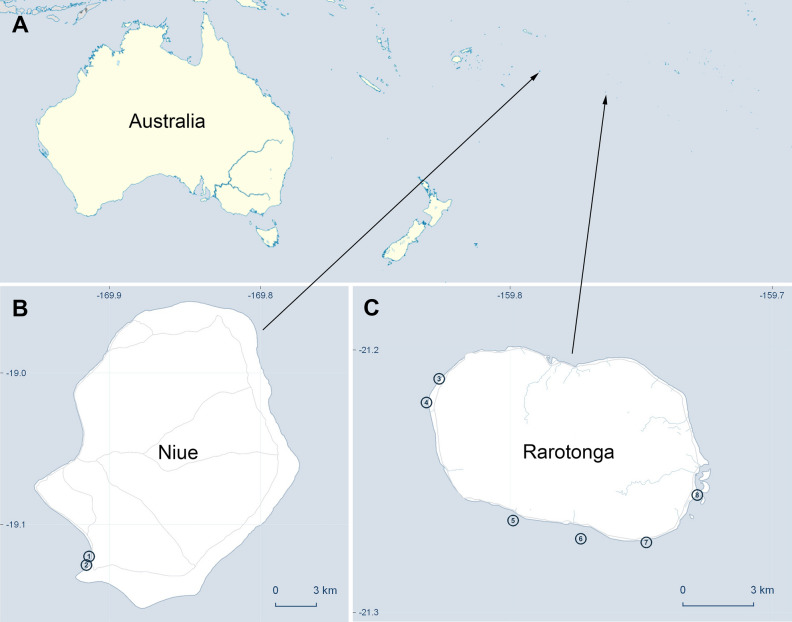
Map of the sampling sites. (**A**) Locations of Niue Island and Rarotonga, Cook Islands in the South Pacific Ocean. (**B**) Sampling sites at Niue Island (1. Avatele Beach, 2. Tamakautoge Beach). (**C**) Sampling sites Rarotonga, Cook Islands (3. Black Rock, 4. Sheraton Passage, 5. Papua Passage, 6. Queen’s Residence, 7. Titikaveka and 8. Muri Lagoon).

However, there are still several undescribed ribotypes that need to be assigned taxonomically. Amongst them, “*Ostreopsis* sp. 3” was first reported from the Cook Islands in Sato et al.^35^ and more recently in Rhodes et al.^52^ from Rangitāhua Kermadec Islands (an Aotearoa New Zealand territory), 1000 km northeast of Aotearoa New Zealand^35,52^. The central aim of this study is to investigate “*Ostreopsis* sp. 3” from the location where it was first reported, the Cook Islands, and other strains isolated from Niue, also located in the South Pacific Ocean (Fig. 1). In November 2014 and several subsequent expeditions, comprehensive sampling of benthic dinoflagellates from macroalgal substrates was carried out at selected lagoon sites around Rarotonga, Cook Islands and Niue. Clonal cultures of *Ostreopsis* spp. were established and assessed using microscopy analysis and molecular data derived from large subunit ribosomal DNA (LSU rDNA; D1-D3 and D8-D10 regions) and internal transcribed spacer regions and 5.8S rRNA gene (ITS/5.8S rDNA) regions. In addition, co-occurring *O. lenticularis*, *C. malayensis* and *C. tropicalis* were also identified and described from these samples. The toxin production of these isolates was investigated using liquid chromatography-tandem mass spectrometry (LC–MS/MS). *Ostreopsis* strains were screened for PLTX-like compounds and *Coolia* strains were screened for gambierone and 44-methyl gambierone (44-MG). Altogether, the morphological, phylogenetic, and toxicological data allowed to describe “*Ostreopsis* sp. 3” as a new species, *Ostreopsis tairoto* sp. nov, and updated the current knowledge of biodiversity and distribution of potentially toxic benthic dinoflagellates in sub-tropical reef systems.

## Results

Twelve *Ostreopsis* and six *Coolia* isolates were established in this study (Tables 1 and 2). Six *Ostreopsis* isolates were identified as the previously described *Ostreopsis lenticularis*, which is the first report and description from the Cooks Islands. Morphological features of these strains are comparable to those previously described for this species. Five strains of *Coolia tropicalis* and one strain of *C. malayensis* are also described in this study. Furthermore, strains of a previously reported ribotype, “*Ostreopsis* sp. 3” were isolated from Cook Islands and Niue and named *Ostreopsis tairoto* sp. nov. based on support of molecular and morphological data.

**Table 1:** Summary of the *Ostreopsis* strains sampled in this study.

S. no	Strain name	Species	Collection site	Country	GPS coordinates	Isolation date	Macroalgae sampled	Temp (°C)	Sal	PLTX-like compounds (pg/cell)
1	O1C6	*Ostreopsis tairoto*	Titikaveka beach	Rarotonga, Cook Islands	21°16′24″ S 159°44′53″ W	November 2014	*Halimeda* sp.	27.2	33.4	< LOD
2	CAWD 268		Arorangi district	Rarotonga, Cook Islands	21°13′12″ S 159°49′55″ W	June 2017	*Halimeda* sp.	*na*	*na*	< LOD
3	CAWD 270		Arorangi district	Rarotonga, Cook Islands	21°13′12″S 159°49′55″ W	June 2017	*Halimeda* sp.	*na*	*na*	< LOD
4	CAWD 287		Avatele	Niue	19°07′36.1″ S 169°54′49.3″ W	September 2018	*Halimeda* sp.	*na*	*na*	< LOD
5	CAWD 311		Tamakautoge	Niue	19°07′36.1″S 169°54′49.3″ W	July 2019	*Halimeda* sp.	*na*	*na*	< LOD
6	CAWD 312		Tamakautoge	Niue	19°07′36.1″S 169°54′49.3″ W	July 2019	*Halimeda* sp.	*na*	*na*	< LOD
7	C209	*Ostreopsis lenticularis*	Muri Lagoon	Rarotonga, Cook Islands	21°15′19″S 159°43′43″ W	November 2014	*Padina* sp.	27.7	34.1	< LOD
8	C202		Muri Lagoon	Rarotonga, Cook Islands	21°15′19″ S 159°43′43″ W	November 2014	*Padina* sp.	27.7	34.1	< LOD
9	S8		Muri Lagoon	Rarotonga, Cook Islands	21°15′19″ S 159°43′43″ W	November 2014	*Padina* sp.	27.7	34.1	< LOD
10	S8Ig9		Muri Lagoon	Rarotonga, Cook Islands	21°15′19″ S 159°43′43″ W	November 2014	*Padina* sp.	27.7	34.1	< LOD
11	CAWD 239		Arorangi district	Rarotonga, Cook Islands	21°13′12″ S 159°49′55″ W	April 2017	*Halimeda* sp.	*na*	*na*	< LOD
12	CAWD 266		Arorangi district	Rarotonga, Cook Islands	21°13′12″ S 159°49′55″ W	April 2017	*Halimeda* sp.	*na*	*na*	< LOD

*na* represents no data, LOD represents limit of detection of the LC–MS/MS method used for screening PLTX-like compounds.

**Table 2:** Summary of the *Coolia* strains sampled in this study.

S. no	Strain name	Species	Collection site	Country	GPS coordinates	Isolation date	Macroalgae sampled	Temp (°C)	Sal	Gambierone (pg/cell)	44-methyl gambierone (pg/cell)
1	C11C2	*Coolia tropicalis*	Sheraton Passage	Rarotonga, Cook Islands	21°12′40″ S 159°49′33″ W	November 2014	*Padina* sp.	26.8	34.3	< 0.05	10
2	C7C1		Queens Residence	Rarotonga, Cook Islands	21°16′19″ S 159°46′23″ W	November 2014	*Padina* sp.	27.9	33.1	*na*	< 0.05
3	C10C1		Papua Passage	Rarotonga, Cook Islands	21°15′54″ S 159°47′56″ W	November 2014	*Padina* sp.	25.1	34.1	< 0.05	11
4	C10C2		Papua Passage	Rarotonga, Cook Islands	21°15′54″ S 159°47′56″ W	November 2014	*Padina* sp.	25.1	34.1	< 0.05	8
5	C12C2		Black Rock	Rarotonga, Cook Islands	21°12′40″ S 159°49′33″ W	November 2014	*Padina* sp.	26.8	34.3	< 0.05	8
6	C6C1	*Coolia malayensis*	Queens Residence	Rarotonga, Cook Islands	21°16′19″ S 159°46′23″ W	November 2014	*Padina* sp.	27.9	33.1	*na*	< 0.05

*na* represents no data.

### Morphology of *Ostreopsis* species

#### Description of *Ostreopsis tairoto* sp. nov. Verma, Hoppenrath, Smith, Rhodes & Murray

**Figure 2:**
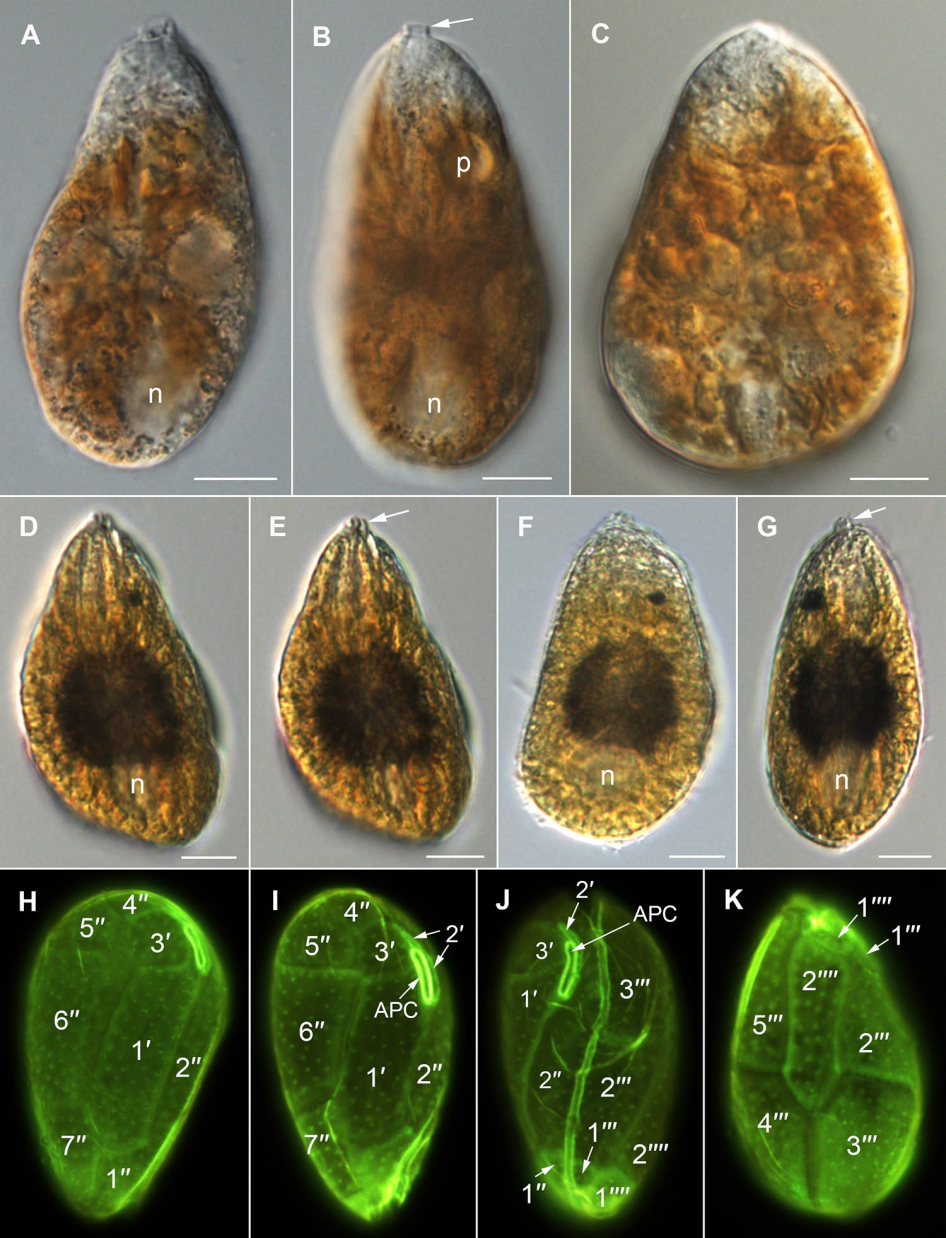
Light micrographs of *Ostreopsis tairoto* sp. nov. strain O1C6 showing the cell shape and general features, including stained thecal plates visualized by epifluorescence. (**A**–**C**) Living cells. (**D**–**G**) Lugol fixed cells. (**H**–**K**) Solophenyl Flavine stained cells viewed by epifluorescence microscopy. (**A**) Mid cell focus, note the dorsal nucleus (n). (**B**) Cell focussed on the ventral opening (arrow) with visible pusule (p) and dorsal nucleus (n). (**C**) Example for a wider ovoid cell. (**D,E**) Same cell in different focal planes. (**D**) Mid cell focus, note the dorsal nucleus (n). (**E**) Cell focussed on the ventral opening (arrow). (**F**) Wider ovoid cell. (**G**) Narrower elongated cell. Note the dorsal nucleus (n) and the ventral opening (arrow). (**H**) Apical view. (**I**) Apical to left lateral view. (**J**) Left lateral view showing the slightly undulating cingulum path. (**K**) Antapical view. Scale bars = 10 µm.

**Figure 3:**
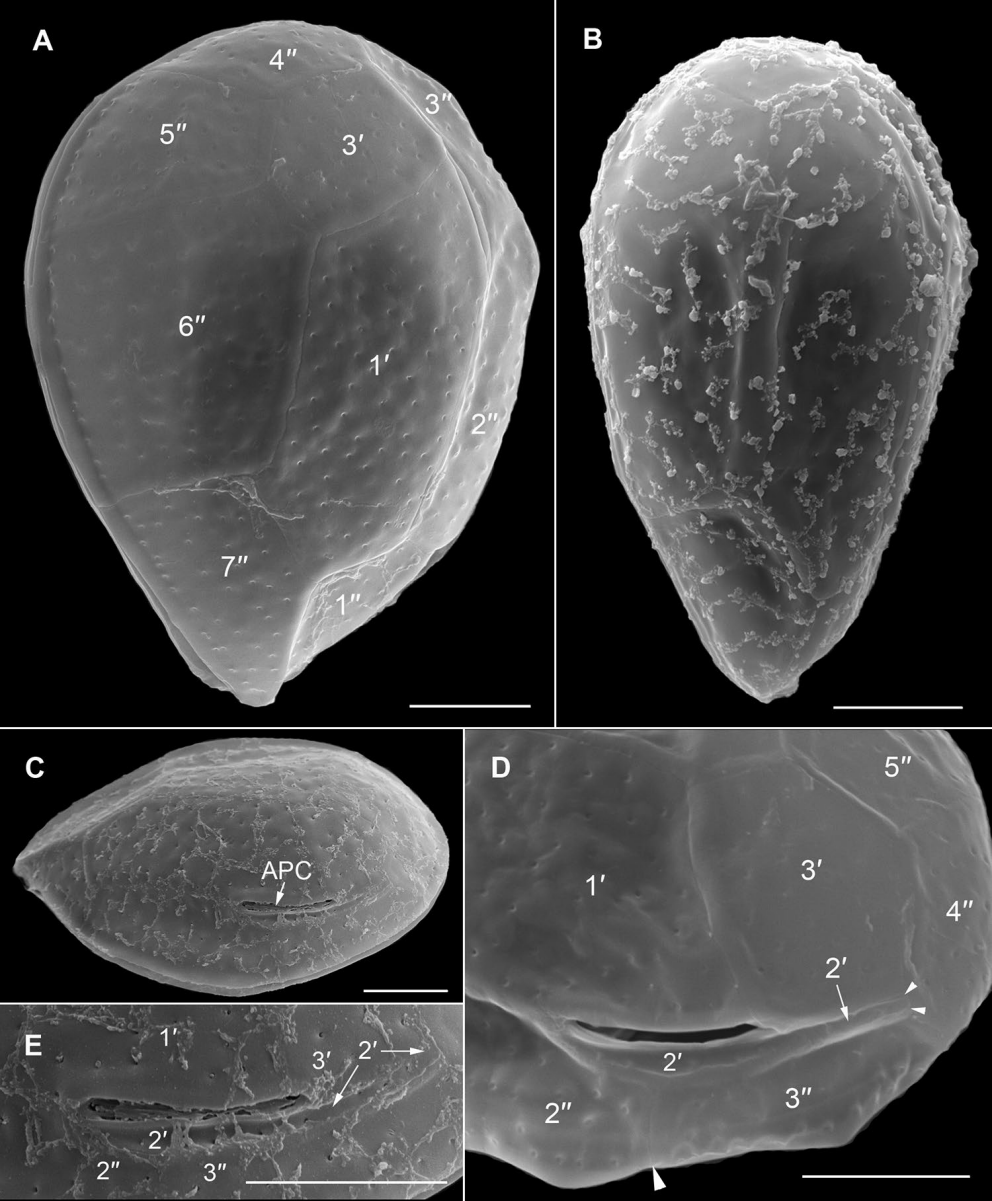
Scanning electron micrographs of *Ostreopsis tairoto* sp. nov. cells from field samples showing the epithecal tabulation. (**A,B**) Apical view of a wide (**A**) and narrow (**B**) cell. (**C**) Left lateral to apical view showing the apical pore complex (APC) in left dorsal position. (**D**) Detail of the left dorsal epitheca with the narrow and elongated second apical plate (2′) below the APC separating the third apical (3′) from the third precingular (3′′) plate and contacting the fourth precingular (4′′) plate. Note the dorsal end of the 2′ plate (small arrowheads) and the suture between plates 2′′ and 3′′ (large arrowhead). (**E**) Detail of the left elongated 2′ plate below the APC separating the 3′ from the 3′′ plate. Scale bars = 10 µm.

**Figure 4:**
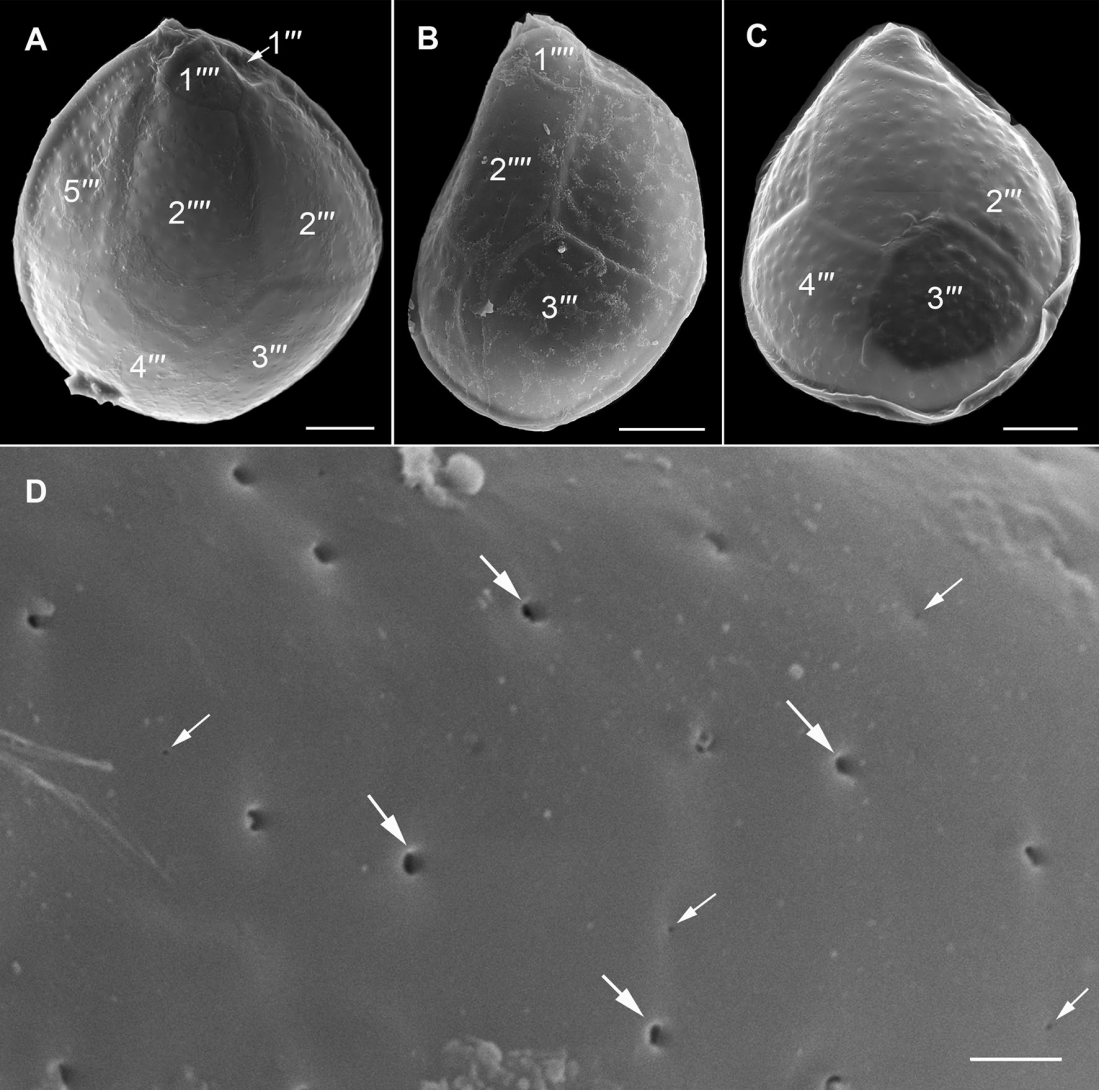
Scanning electron micrographs of *Ostreopsis tairoto* sp. nov. cells from field samples showing the hypothecal tabulation and thecal pores. (**A**) Antapical to ventral view. (**B**) Left lateral to antapical view. (**C**) Antapical to dorsal view. Note the different cell shapes. (**D**) Pores of two size classes, main larger pores (large arrows) and tiny pores (small arrows) only recognizable with very high magnification. Scale bars = 10 µm, except D: 1 µm.

The strongly anterio-posteriorly flattened cells were ovate (drop/tear-shaped) and ventrally tapering (Fig. 2), variable in shape and relative cell width (Figs. 2A–G, 3A,B and 4A–C). Cells were 40–76 µm in dorso-ventral (DV) depth (mean: 54.8 ± 7.9 µm s.d., n = 15 cells each from 3 separate strains) and 21–44 µm wide (W) (mean: 30.6 ± 5.5 µm s.d., n = 15 cells each from 3 separate strains), and varied from 1.33 to 2.6 (mean: 1.8, n = 15 cells each from 3 separate strains) in DV:W ratio (Table 3). Cells contained golden-brown chloroplasts, except for the ventral area (Fig. 2A–C). The nucleus was located dorsally (Fig. 2A,B,D,F,G). Pusules were recorded (Fig. 2B). The plate formula was APC 3′ 7′′ ?c ?s 5′′′ 2′′′′ (Figs. 2H–K, 3 and 4A–C). The narrow, slightly curved and elongated apical pore complex (APC) was located parallel to the left mid-lateral to dorsal cell margin (Figs. 2I,J and 3). The apical or outer pore plate (Po) was about 6.5–9.0 µm long (n = 4), had a slit-like apical pore and less thecal pores in an irregular row below the apical pore and more scattered thecal pores above it (Supplementary Fig. 1E). The first apical plate (1′) was long, hexagonal and located left to the centre of the epitheca (Figs. 2H,I and 3A,B). The characteristic second apical plate (2′) was narrow and elongated, about 1.5 times as long as the Po plate (Fig. 3D,E and Supplementary Fig. 1F). Plate 2′ completely separated plate 3′ from 3′′ (Fig. 3D,E). The third apical plate (3′) was pentagonal, had a suture with plate 6′′, and did not touch plate 3′′ (Fig. 3A,D). In the precingular series plate 1′′ was the smallest and 6′′ the largest (Figs. 2H and 3A,B). All precingular plates were four-sided, except the second (2′′) and sixth (6′′) that were pentagonal (Fig. 3A,D). Plate 5′′ was not in contact with plate 1′ (Fig. 3A,D). The cingulum was narrow, deep, and slightly undulated (Figs. 2J and 3C). Cingular plates could not be documented. The postcingular plate series consisted of a very small first (1′′′) plate, and four large plates (2′′′-5′′′) (Figs. 2K and 4A–C). The two antapical plates were of unequal size, 1′′′′ relatively small and 2′′′′ pentagonal and nearly symmetrical, relatively narrow with nearly parallel sides (sutures with 2′′′ and 5′′′) (Figs. 2K and 4A–C). The sulcal plates could not be determined. Thecal plates were smooth with scattered pores of medium size (0.19–0.27 µm in diameter, n = 20) with an internal sieve-like structure of small pores (Fig. 4D and Supplementary Fig. 1C,D). Only with very high magnification scattered small pores (about 0.06–0.08 µm, n = 5) in lower density were observed (Fig. 4D).

**Table 3:** Morphological comparison between *Ostreopsis* species closely related to *O.* *tairoto*.

Species	*O. tairoto*	*O. fattorussoi*^11^	*O. rhodesiae*^12^	*O.* cf. *ovata*^36,69,71^	*O.* cf. *siamensis*^36,69,71^	*O. mascarenensis*^16^
Depth (DV)	52–70 µm^a^	42.5–72.5 µm	28.3–57.8 µm	55.0–84.0 µm^71^	55.0–74.5 µm^71^	112.0–141.7 µm
	40–70 µm^b^			36.0–60.0 µm^69^	63.0–78.0 µm^69^	
	44–76 µm^c^			27.0–65.0 µm^36^	50.0–90.0 µm^36^	
Width (W)	22–44 µm^a^	26.3–50.0 µm	16.7–44.2 µm	30.0–62.0 µm^71^	27.0–56.0 µm^71^	82.4–108.8 µm
	21–38 µm^b^			24.0–45.0 µm^69^	36.0–54.0 µm^69^	
	24–40 µm^c^			19.0–57.0 µm^36^	34.0–62.0 µm^36^	
DV/W ratio	1.33–2.6^a^	1.52 ± 0.14	1.1–2.1	1.2–1.95	1.1–2.15	1.15–1.44
	1.37–2.22^b^			1.9–2.74	1.3–1.94	
	1.3–2.4^c^					
Po length	6.5–9.0 µm	10.0–12.5 µm	9.0–11.0 µm	9.6–13.5 µm^71^	10.3–11.9 µm^71^	21.1–26.4 µm
				6.3–8.3 µm^69^	11.0–13.0 µm^69^	
				6.9–9.6 µm^36^	7.4–9.7 µm^36^	
Plate 1′	Left to centre hexagonal	Left to centre hexagonal*	Most left to centre hexagonal	Most left to centre hexagonal	Most left to centre hexagonal	~Left to centre hexagonal
Plate 2′	1.5 × Po length	~ 2 × Po length	~ 2 × Po length	1.5–2 × Po^69^/n.d.^36,71^	~ 1.5 × Po length	1.6–2 × Po length
Plate 3′	No contact with 3′′	No contact with 3′′	No contact with 3′′	No^69^ contact with 3′′	No^69^ contact with 3′′	No contact with 3′′
				Contact with 3′′ ^#^	No^##^ contact with 3′′	
	Suture with 6′′	Suture with 6′′	Suture with 6′′	Suture with 6′′	Suture with 6′′	Suture with 6′′
	Penta/hexagonal	Penta/hexagonal**	Penta/hexagonal**	Penta/hexagonal	Penta/hexagonal	Hexagonal
5′′ contact 1′	no	no	no (few exceptions)	no	no	no (few exceptions)
Plate 6′′	6′′/5′′ suture longer as 6′′/7′′ suture	6′′/5′′ suture longer as 6′′/7′′ suture	6′′/5′′ suture longer as 6′′/7′′ suture	6′′/5′′ suture longer as 6′′/7′′ suture	6′′/5′′ suture longer as 6′′/7′′ suture	6′′/5′′ suture longer as 6′′/7′′ suture
Cingulum	Slightly undulated	n.d	Slightly undulated	Straight	Straight	Slightly undulated
					Slightly undulated^69^	
Plate 2′′′′	Nearly symmetrical	Asymmetrical	Asymmetrical	Asymmetrical	Asymmetrical	Asymmetrical
	Relatively narrow	Relatively narrow	Relatively narrow	Relatively narrow	Narrow	Relatively narrow
	Nearly parallel sides	Nearly parallel sides	Nearly parallel sides	Parallel sides	Parallel sides	Nearly parallel sides
Thecal pores						
Small	0.06–0.08 µm	none	none	None^36,69,71^	0.07–0.13 µm^71^	0.05–0.07 µm
Large	0.19–0.27 µm	0.26–0.53 µm	0.16–0.30 µm^§^	0.16–0.55 µm^36^	0.11–0.56 µm^36^	0.30–0.40 µm
				0.12–0.25 µm^69^	0.14–0.32 µm^69^	
				0.24–0.56 µm^71^	0.15–0.39 µm^71^	

^a,b,c^represent DV, W and DV/W ratio measurements obtained from *O. tairoto* strains O1C6, CAWD268 and CAWD270 respectively.

**Figure 5:**
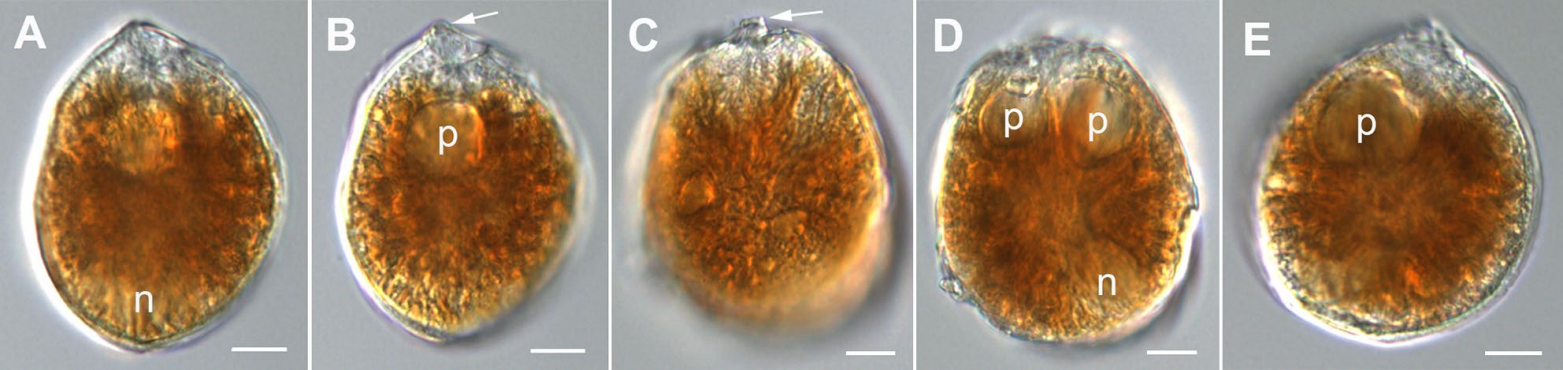
Light micrographs of *Ostreopsis lenticularis* strain C202 showing the cell shape and general features. (**A,B**) Same drop-shaped cell in different focal layers. Note nucleus (n), pusule (p) and ventral opening (arrow). (**C,D**) Same broadly oval to ovoid cell in different focal layers. Note the ventral opening (arrow) in C and the nucleus (n) and two pusules (p) in (**D**). (**E**) A relatively round cell with pusule (p). Scale bars = 10 µm.

*described as heptagonal but hexagonal on Figs. 2A, 4A and 5A (Accoroni et al.^11^); **counting sutures it is hexagonal but as the very short suture with the Po plate is not recognized in lower magnification it looks pentagonal on most images; ^#^David et al.^69^: contact described in text, but see their Fig. 6A; ^##^David et al.^69^: contact described in text, but see their Figs. 3A,D and 4A and Penna et al.^36^ Fig. 3A. ^§^measured on original SEMs (n = 40) from Verma et al.^12^.

*Holotype:* SEM-stub (designation CEDiT2022H147) prepared from strain O1C6 and deposited at Senckenberg am Meer, German Centre for Marine Biodiversity Research, Centre of Excellence for Dinophyte Taxonomy, Germany. Cells from the holotype are shown in Supplementary Fig. 1.

*Reference material:* Lugol-fixed subsample of strain O1C6 (designation CEDiT2022I148) deposited at the Senckenberg am Meer, German Centre for Marine Biodiversity Research, Centre of Excellence for Dinophyte Taxonomy, Germany.

*Type locality:* Titikaveka Beach (21°16′24" S; 159°44′53" W) Rarotonga, Cook Islands.

*Etymology: Tairoto* is the Cook Islands Māori term for ‘from/of a wide lagoon’, i.e. isolated/ inhabitant of sea inside the lagoons of Rarotonga, Cook Islands, which is the type locality of this species.

#### *Ostreopsis lenticularis* Y.Fukuyo

**Figure. 6:**
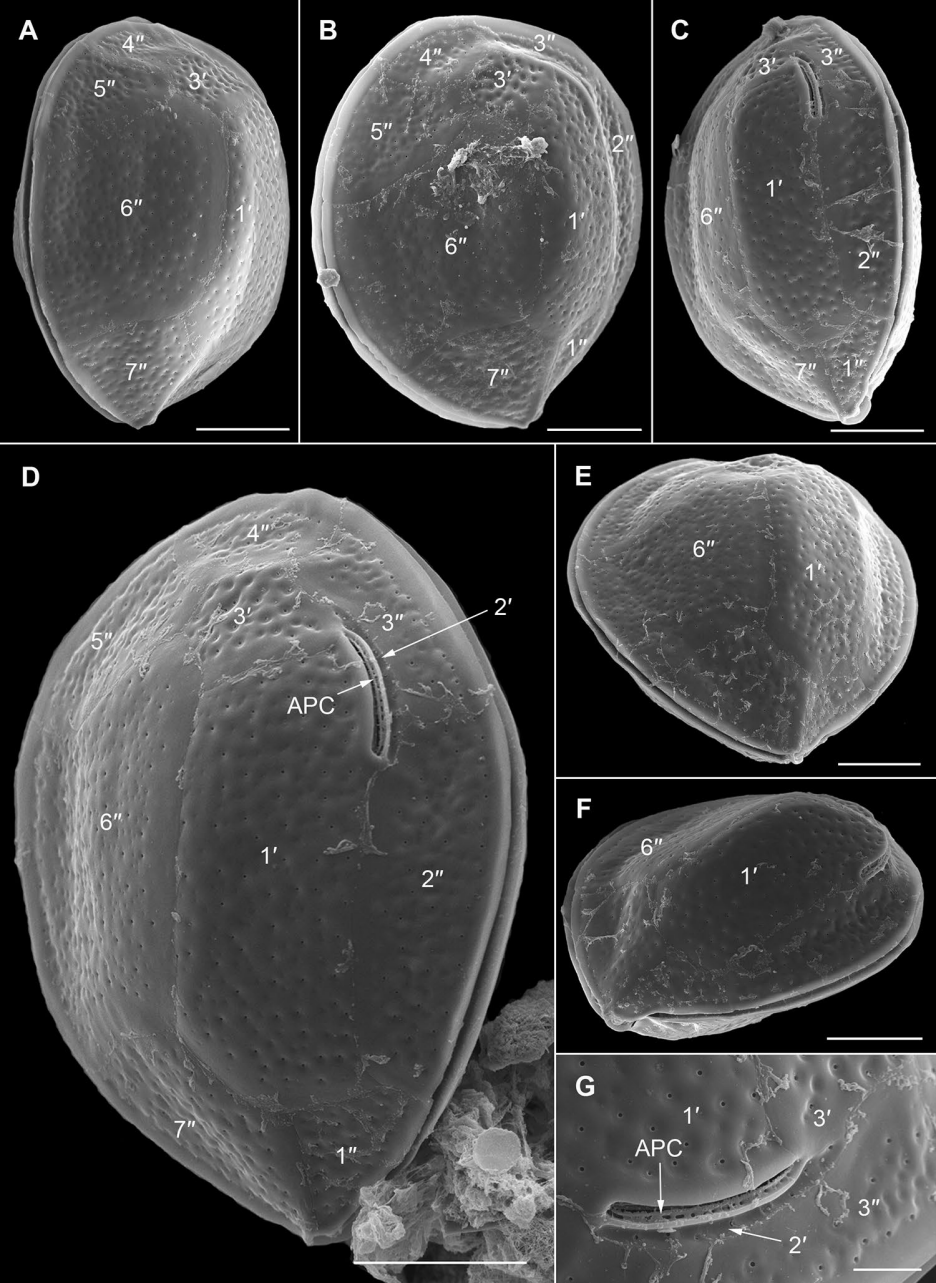
Scanning electron micrographs of *Ostreopsis lenticularis* cells from field samples showing the epithecal tabulation. (**A**) Apical to right lateral view. (**B**) Apical view. (**C,D**) Apical to left lateral view. (**E**) Ventral to apical view. (**F**) Left lateral to ventral view. (**G**) Detail of the apical pore complex (APC). Scale bars = 20 µm, except G: 5 µm.

**Figure 7:**
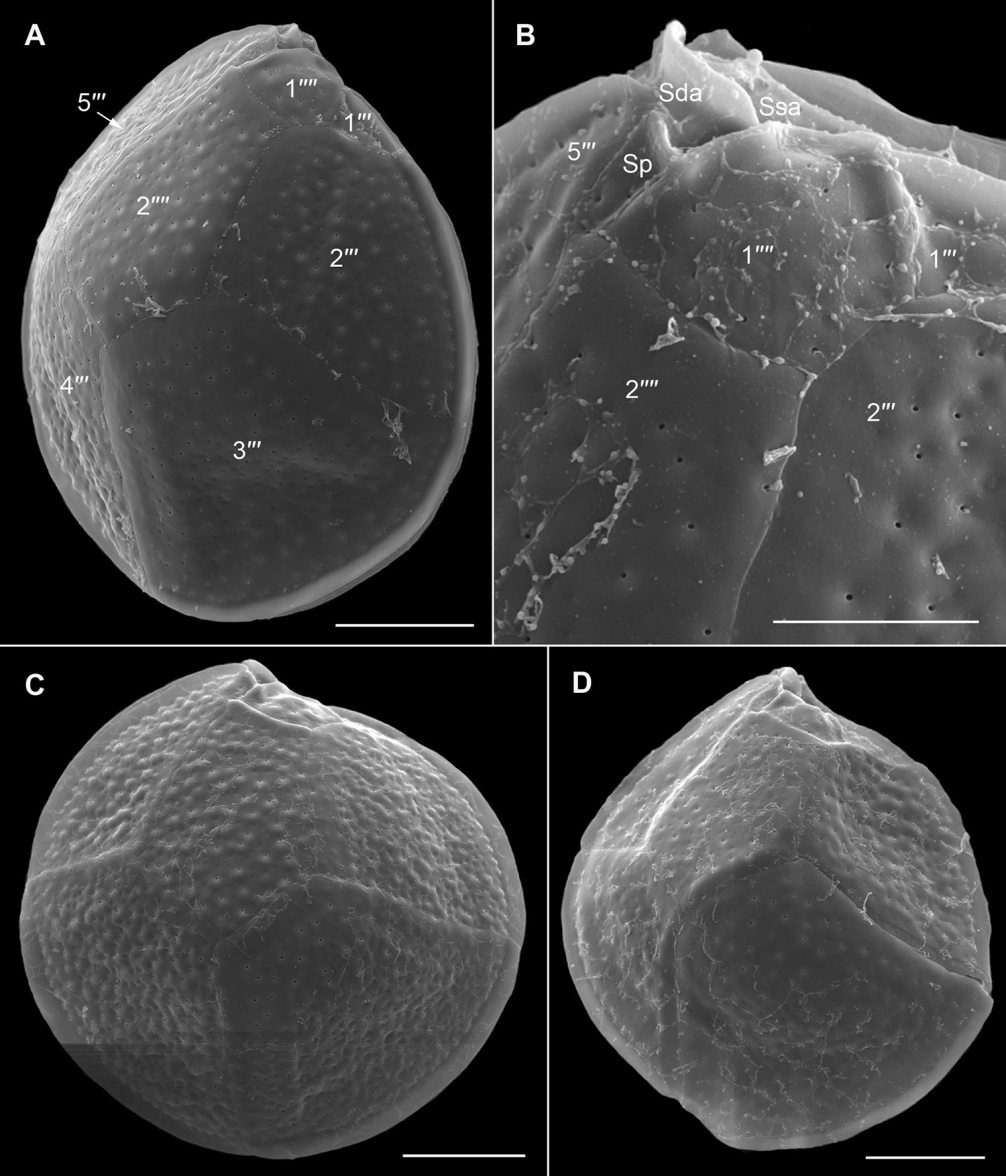
Scanning electron micrographs of *Ostreopsis lenticularis* cells from field samples showing the hypothecal tabulation. (**A**) Left lateral to antapical view. (**B**) Detail of the ventral area showing three sulcal plates, the posterior (Sp), the anterior right (Sda) and the anterior left (Ssa) sulcal plate. (**C,D**) Antapical view, note the different cell shapes. Scale bars = 20 µm, except B: 10 µm.

**Figure 8:**
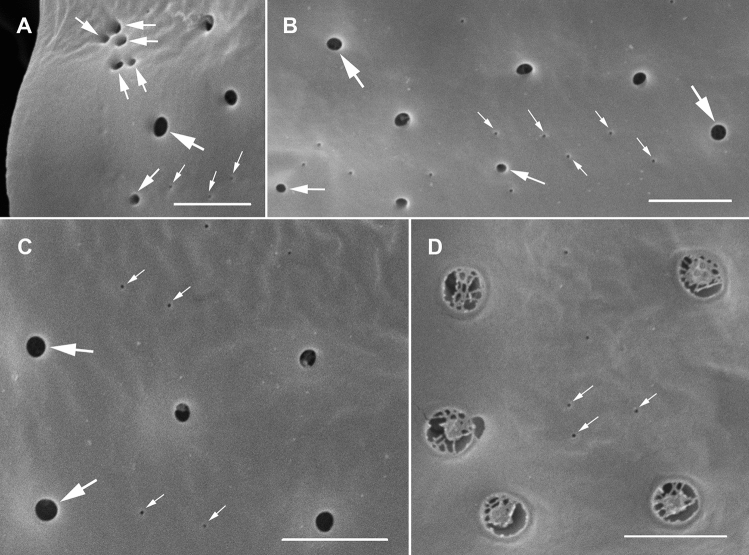
Scanning electron micrographs of *Ostreopsis lenticularis* strain C202 showing the thecal pores. (**A–C**) Outside views showing large (large arrows), medium sized (medium arrows in A and B) and small (small arrows) pores. Note the group of medium pores close to the plate margin in A. (**D**) Inside view, recognize the inner covering perforated by irregular openings of large pores and uncovered small pores (small arrows). Scale bars = 2 µm.

The photosynthetic cells were anterio-posteriorly flattened, lenticular, broadly ovate (drop-shaped) to almost round and ventrally tapering in apical and antapical views (Fig. 5). The field samples measured 78.1–87.5 μm in depth (n = 12), and 55.5–82.9 µm in width (n = 12). The mean DV:W ratio was 1.24 (1.0–1.57, n = 12). The nucleus was located dorsally (Fig. 5A,D). One or two pusules were recorded (Fig. 5B,D,E). The thecal plate pattern was: APC 3′ 7′′ ?c ?s 5′′′ 2′′′′ (Figs. 6 and 7). The slightly curved and elongated apical pore complex (APC) was located parallel to the left dorsal cell margin (Fig. 6C,D,G). The apical (or outer) pore plate (Po) was about 11.5–18.0 µm long (n = 7). The first apical plate (1′) was long, hexagonal and most of it located left to the centre of the epitheca (Fig. 6B–F). The second apical plate (2′) was narrow and slightly longer as the Po plate (Fig. 6D,G). The third apical plate (3′) had a short suture with the Po plate (Fig. 6B–D,G). In the precingular series plate 1′′ was the smallest and 6′′ the largest (Fig. 6A–F). The suture between plates 2′′ and 3′′ was located at about half Po plate length (not shown). Plate 5′′ was not in contact with plate 1′ (Fig. 6A,B,D). The cingulum was narrow, deep, and not undulated (Fig. 6A,C–F). Cingular and most sulcal (Fig. 7B) plates were not observed. The postcingular plate series consisted of a small first (1′′′) plate and four large plates (2′′′-5′′′) (Fig. 7A,C,D). The two antapical plates were of unequal size, 1′′′′ smaller and 2′′′′ being asymmetrical and relatively large (Fig. 7A,C,D). The left side of the posterior sulcal plate (Sp) was in contact with the 1′′′′ plate and its posterior end with the 2′′′′ plate (Fig. 7B). Thecal plates were smooth with scattered large pores (0.35–0.51 µm in diameter, n = 20) and small pores (0.06–0.08 µm in diameter, n = 19) (Fig. 8A–C). Large thecal pores had an inner covering perforated by irregular openings, also known as internal sieve-like structure (Fig. 8D). A few irregularly scattered pores of a medium size (0.20–0.29 µm in diameter, n = 20) were recorded (Fig. 8A,B), in one case arranged in a cluster (Fig. 8A).

### Morphology of *Coolia* species

#### *Coolia malayensis* Leaw, P.T.Lim & Usup

**Figure 9:**
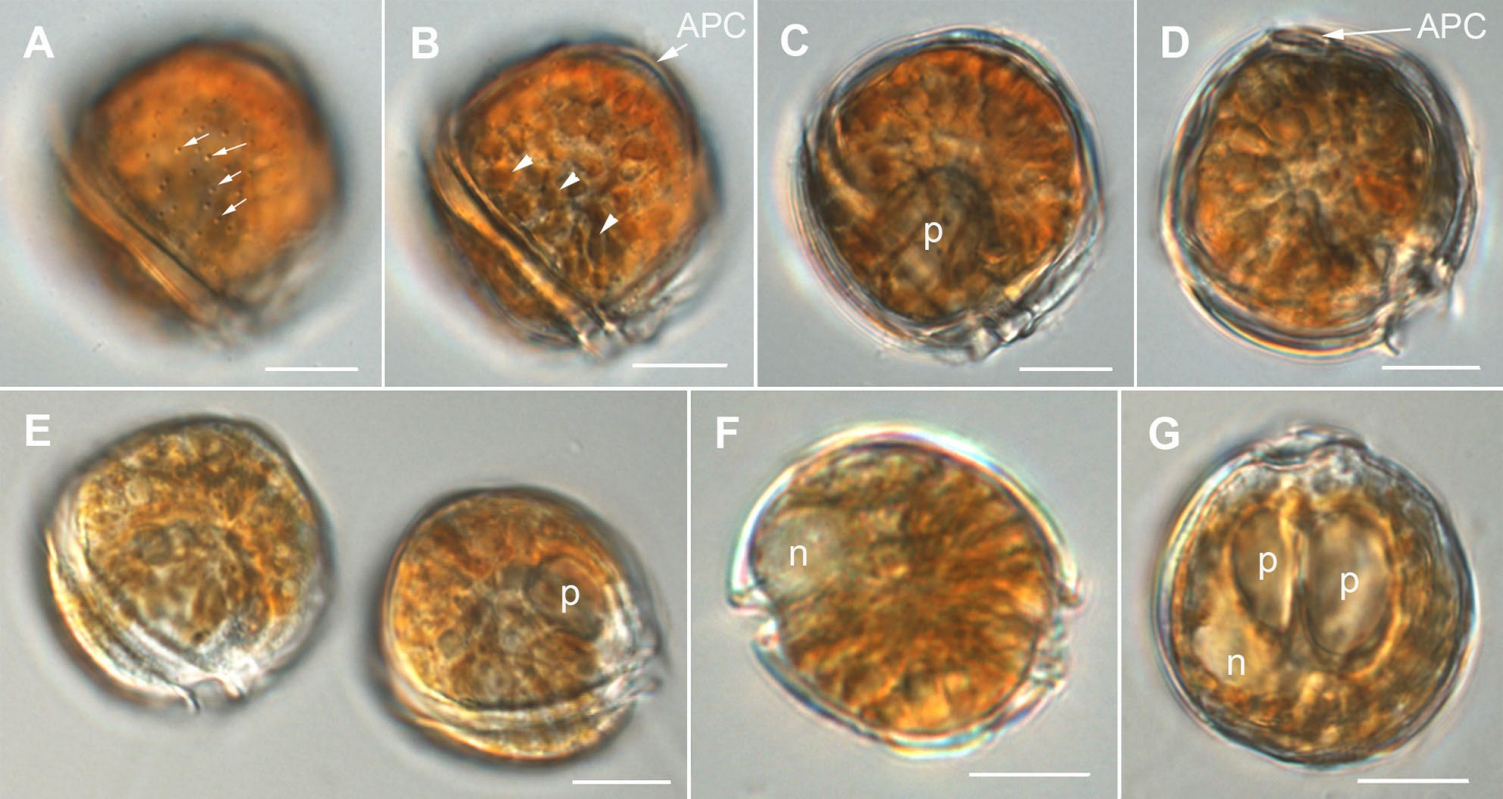
Light micrographs of *Coolia malayensis* strain C6C1 showing the cell shape and general features. (**A–C**) Same cell in different focal planes. Globular cell, ventrally slightly tapering and containing chloroplasts. (**A**) Surface focus on the epitheca showing thecal pores (arrows). (**B**) The slit-like apical pore complex (APC) can be recognized; chloroplasts are distinguishable (arrowheads). (**C**) Mid cell focus showing the pusule (p). (**D**) Apical view showing the APC in dorsal left position. (**E**) Two specimens in different views demonstrating the spherical cell shape. Note the pusule (p) in the right cell. (**F**) Right lateral view, mid cell focus. The nucleus (n) is located dorsally in the episome. (**G**) Cell in apical or antapical view in mid cell focus showing the nucleus (n) and two pusules (p). Scale bars = 10 µm.

**Figure. 10:**
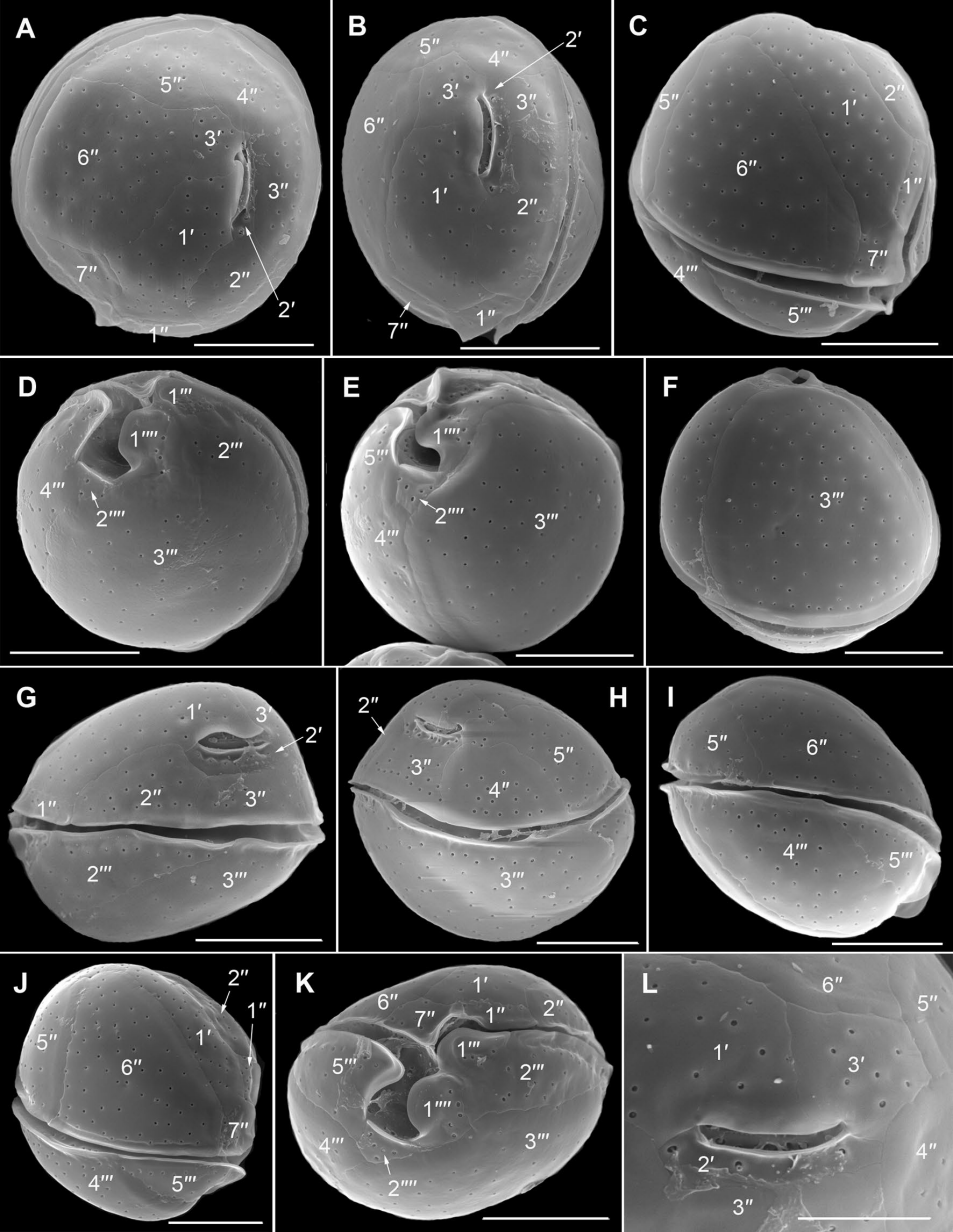
Scanning electron micrographs of *Coolia malayensis* strain C6C1 showing the thecal tabulation. (**A**) Epitheca in apical view. (**B**) Epitheca in apical to left lateral view. (**C**) Ventral to right lateral view. (**D,E**) Hypotheca in antapical view. (**F**) Hypotheca in left lateral view. (**G**) Cell in left lateral view. (**H**) Cell in dorsal view. (**I**) Cell in right lateral view. (**J**) Cell in right lateral to ventral view. (**K**) Cell in ventral view. (**L**) Detail of the dorsal epitheca showing the slit-like apical pore complex surrounded by the apical plates (1–3′). Scale bars = 10 µm, except L: 5 µm.

**Figure 11:**
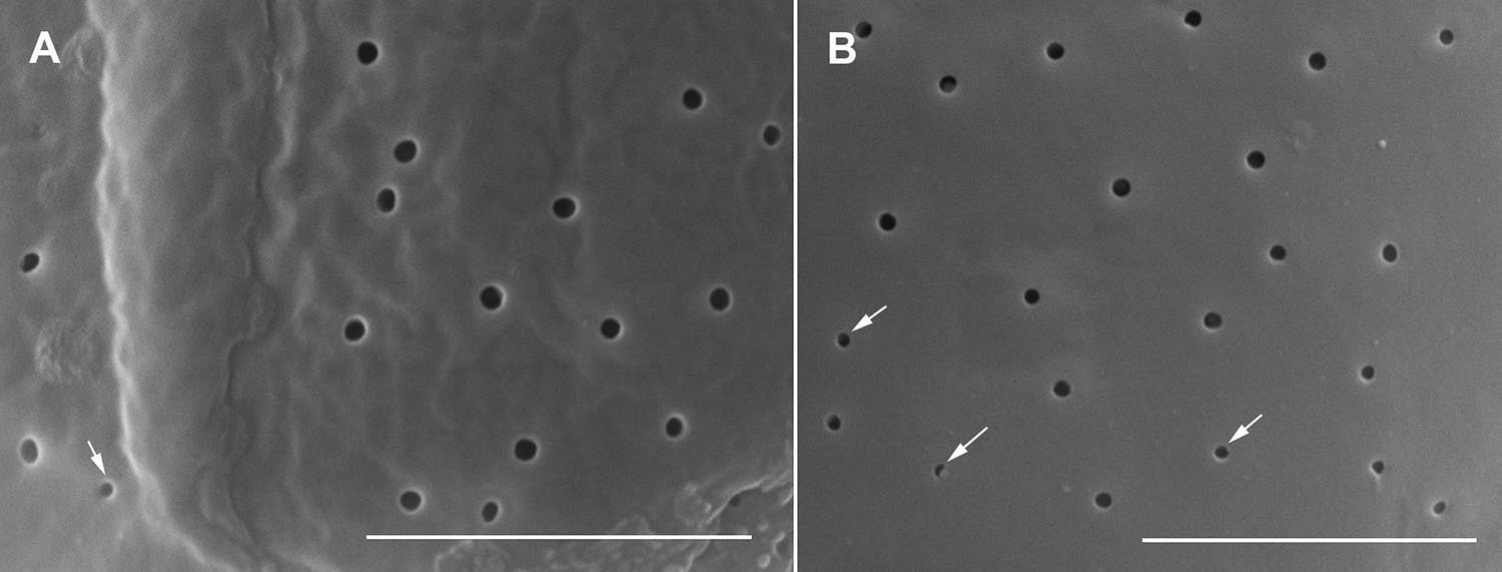
Scanning electron micrographs of *Coolia malayensis* strain C6C1 showing the thecal pores. (**A**) Recognize the plate overlap area without pores. (**B**) Large and slightly smaller (arrows) pores are visible. Scale bars = 5 µm.

The cells were globular (Fig. 9), 22.5–30 μm long (mean: 26.8 ± 2.9 μm s.d., n = 12), 18.5–24.6 μm wide (mean: 22.3 ± 2.5 μm s.d., n = 12), and 16.2–25.9 μm deep (mean: 21.8 ± 1.9 μm s.d., n = 8); containing many chloroplasts (Fig. 9B). One or two pusules can be present (Fig. 9C,E,G). Nucleus located in the dorsal episome (Fig. 9F). The thecal plate pattern was: APC 3′ 7′′ ?c ?s 5′′′ 2′′′′ (Fig. 10). The first apical plate (1′) was narrow oblong and located on the left epitheca side (Fig. 10A–C). The third apical plate (3′) was pentagonal (Fig. 10A,B,L) with wide contact (suture) to plate 5′′. The sixth precingular plate (6′′) was the widest and largest plate in the epitheca (Fig. 10A,C,I,J). The seventh precingular plate (7′′) was small and pentagonal (Fig. 10C,J,K). The length of the apical pore plate was about 5.0–6.4 µm (n = 5). Narrow, descending cingulum (Fig. 10G–K). Plate 2′′′′ was in contact with 1′′′′ but not with 2′′′ (Fig. 10D,E,K). The short sulcus was bordered by sulcal lists extending from plates 5′′′, 2′′′′, 1′′′′ and 1′′′ (Fig. 10K). Thecal plates were smooth with scattered round (to oval) thecal pores (Fig. 11A). Most pores were 0.22–0.33 µm in diameter (n = 20), but few pores were slightly smaller, 0.16–0.20 µm (n = 8) in diameter (Fig. 11B).

#### *Coolia tropicalis* M.A.Faust

**Figure 12:**
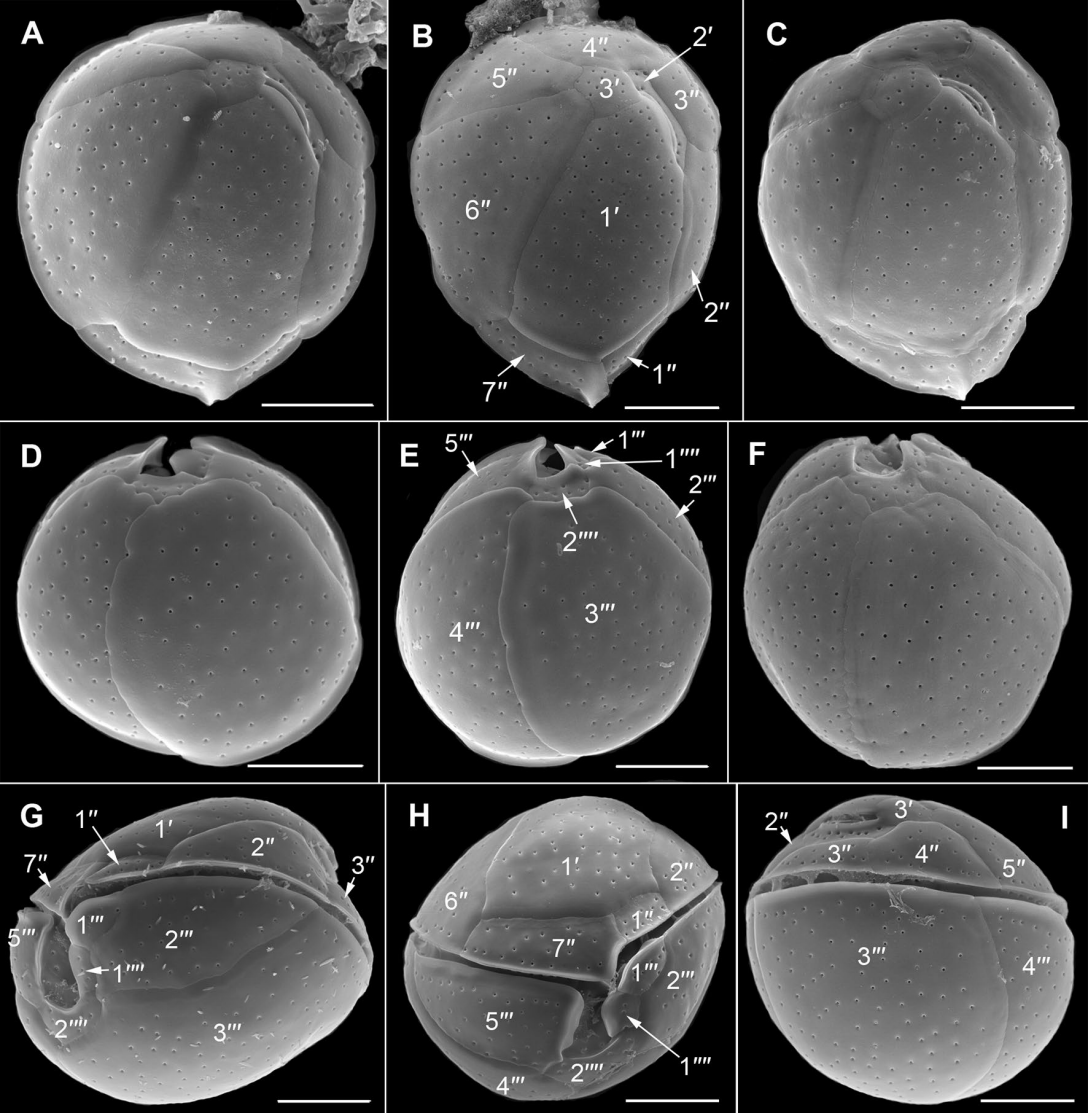
Scanning electron micrographs of *Coolia tropicalis* strain C11C2 showing the thecal tabulation. (**A–C**) Apical view of the epitheca. (**D–F**) Antapical view of the hypotheca. (**A-F**) Note the different shapes partly depending on the growth band development. (**G**) Left lateral to ventral view. (**H**) Ventral view. (**I**) Dorsal view. Scale bars = 10 µm.

**Figure 13:**
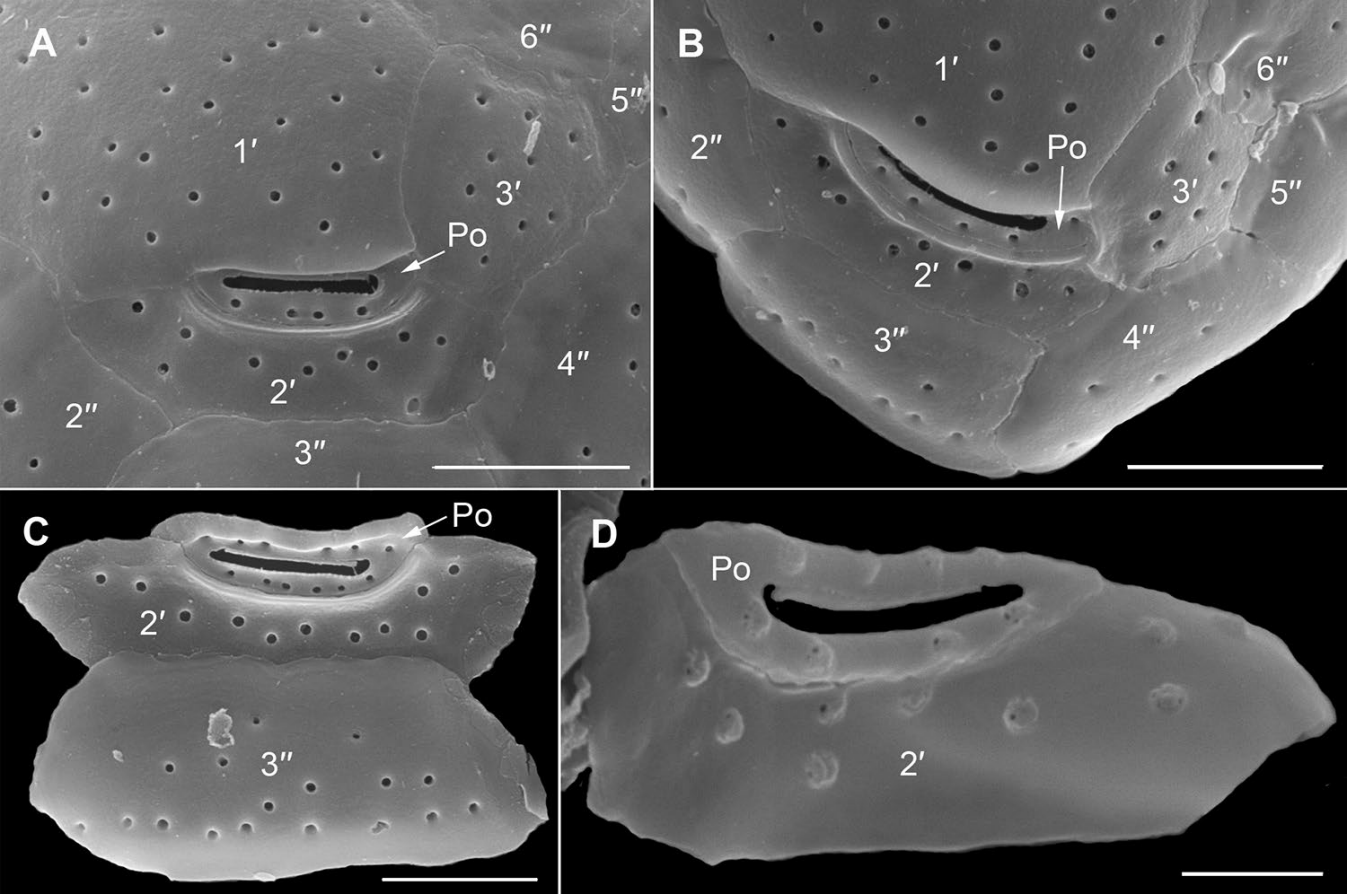
Scanning electron micrographs of *Coolia tropicalis* strain C11C2 showing details of the apical pore complex and surrounding epithecal plates. (**A,B**) Dorsal part of the epitheca showing the apical pore plate (Po) with nearly straight slit-like apical pore. (**C**) Separated epithecal plates, including the Po plate with round thecal pores surrounding the slit-like apical pore. (**D**) Inside view the apical pore plate (Po) with inner covering of the thecal pores perforated by tiny irregular openings. Scale bars = 5.0 µm, except D: 2 µm.

The cells were globular to subspherical, 35.3–51.2 μm (mean: 41.8 ± 6.0 μm s.d., n = 15) long, 32.5–40.2 μm (mean: 36.1 ± 2.8 μm s.d., n = 15) wide, and 26.2–29.1 μm (mean: 27.9 ± 1.3 μm s.d., n = 8) deep; containing chloroplasts (not shown). The thecal plate pattern was: APC 3′ 7′′ ?c ?s 5′′′ 2′′′′ (Fig. 12). The first apical plate (1′) was large, widening ventrally and located centrally on the ventral epitheca side (Fig. 12A–C). The sixth precingular plate (6′′) was nearly the same size as plate 1′ (Fig. 12A,B). The seventh precingular plate (7′′) was pentagonal, large and wide (Fig. 12A–C,H). Plate 2′′′′ was in contact with 1′′′′ and 2′′′ (Fig. 12D–H). Narrow, descending cingulum (Fig. 12G–I). The short sulcus was bordered by sulcal lists extending from plates 5′′′, 2′′′′, 1′′′′ and 1′′′ (Fig. 12D–H). Thecal plates were smooth with scattered round thecal pores (Figs. 12 and 13). Most pores were 0.25–0.38 µm in diameter (n = 14), but very few pores were slightly smaller, 0.19–0.24 µm (n = 5) in diameter (Fig. 13). The length of the apical pore plate (Po) was about 5.7–7.3 µm (n = 6) and of the apical pore about 3.7–5.1 µm (n = 6). The Po plate had round thecal pores surrounding the slit-like apical pore (Fig. 13A–C). Thecal pores had an inner covering perforated by tiny irregular openings, equivalent to internal sieve-like structure of small pores described for other species (Fig. 13D).

### Genetic divergence and molecular phylogeny

**Figure 14:**
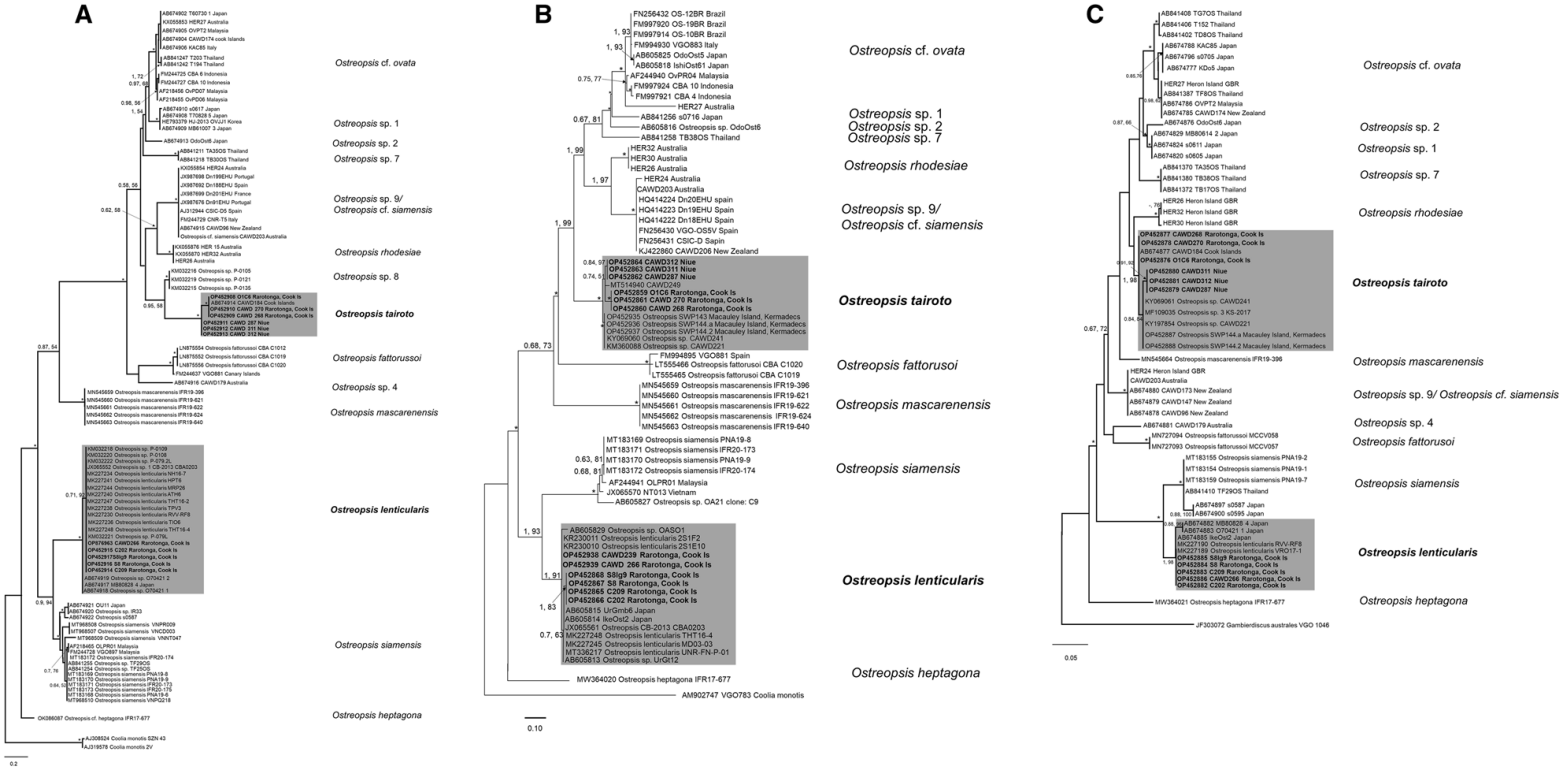
Maximum Likelihood (ML) phylogenetic tree of various *Ostreopsis* strains using primer sets for (**A**) ITS/5.8S rDNA, (**B**) D1-D3 LSU rDNA and (**C**) D8-D10 LSU rDNA. Numbers at nodes represent posterior probabilities from Bayesian Inferences (BI) and bootstrap support values from Maximum Likelihood (ML) based on 1000 pseudo-replicates. *represents 1, 100 support values for BI and ML respectively. Strains isolated is this study are indicated in bold and a grey background.

**Figure 15:**
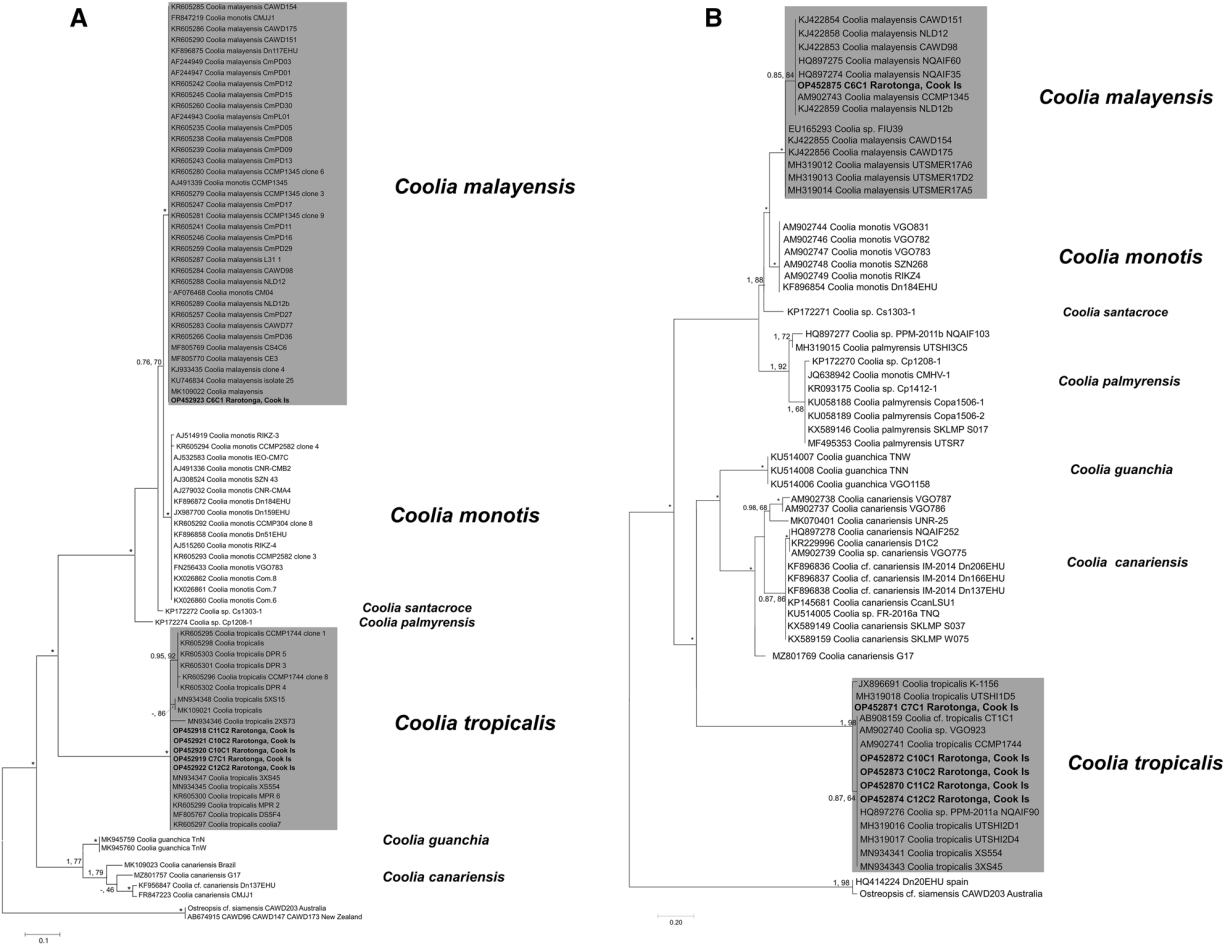
Maximum Likelihood (ML) phylogenetic tree of various *Coolia* strains using primer sets for (**A**) ITS/5.8S and (**B**) LSU D1-D3 rDNA. Numbers at nodes represent posterior probabilities from Bayesian Inferences (BI) and bootstrap support values from Maximum Likelihood (ML) based on 1000 pseudo-replicates. *represents 1, 100 support values for BI and ML respectively. Strains isolated is this study are indicated in bold and a grey background.

ML and BI phylogenetic analyses were performed on alignments of *Ostreopsis* strains isolated in this study, with additional reference sequences from Genbank, and identified 14, 12 and 13 strongly supported clades based on ITS/5.8S, D1-D3 and D8-D10 rDNA analyses respectively (Fig. 14A–C). Six strains of *Ostreopsis tairoto* clustered together and formed a fully supported monophyletic clade (BI = 1.00; ML = 100) (Fig. 14A–C). Both ML and BI analyses gave the same tree topology and identical relationships among *Ostreopsis* clades. Hence, only the majority-rule consensus tree of the ML analysis is shown (Fig. 14A–C). Based on the ITS/5.8 s rDNA analysis, the species is most closely related to *Ostreopsis* sp. 8, with the genetic* p* distance between them calculated as 19.0 ± 3.2%. This species also appears to be closely related to *Ostreopsis mascarenensis*, “*O*. sp. 4”, *O. fattorussoi, O. rhodesiae* and *O.* cf. *siamensis* (Table 4 and Fig. 14A), which is based on both ITS/5.8 s as well as the LSU rDNA analyses. The genetic *p* distance between the *O. tairoto* isolates, that was used in the phylogenetic analyses varied from 4.8 ± 1.2, 2.4 ± 0.5 to 0.3 ± 0.2% in ITS/5.8S, D1-D3 and D8-D10 rDNA analyses respectively (Table 4). The strains isolated from Niue formed a separate sub-clade within *O. tairoto* exhibiting minor genetic variation compared to the Cook Islands strains. In addition, six *O. lenticularis* strains were identified and grouped with sub-clade I of *O. lenticularis* which are described in Chomérat et al.^18^ (Fig. 14A–C). Also, five strains of *Coolia tropicalis* and one strain of *Coolia malayensis* were identified based on ITS/5.8S and D1-D3 LSU regions (Fig. 15A,B).

**Table 4:** Distance values (pairwise uncorrected *p* distances) based on the ITS/5.8S, D1-D3, D8-D10 LSU rDNA sequences respectively within and between *Ostreopsis* species closely related to *O.* *tairoto*.

Species	*O. tairoto*	*O. mascarenensis*	*O. fattorussoi*	*Ostreopsis* sp. 8	*Ostreopsis* sp. 4	*O. rhodesiae*	*Ostreopsis* sp. 9/*O.* cf. *siamensis*
*O. tairoto*	0.048 (0.012)						
	0.024 (0.005)						
	0.003 (0.002)						
*O. mascarenensis*	0.582 (0.034)	0.000 (0.000)					
	0.329 (0.017)	0.000 (0.000)					
	0.036 (0.006)	*na*					
*O. fattorussoi*	0.328 (0.028)	0.534 (0.033)	*–*				
	0.344 (0.021)	0.32 (0.021)	0.026 (0.007)				
	0.05 (0.008)	0.052 (0.008)	0.000 (0.000)				
*Ostreopsis* sp. 8	0.19 (0.032)	0.581 (0.035)	0.325 (0.033)	*na*			
	*na*	*na*	*na*	*na*			
	*na*	*na*	*na*	*na*			
*Ostreopsis* sp. 4	0.318 (0.033)	0.571 (0.027)	0.251 (0.031)	0.319 (0.032)	*na*		
	*na*	*na*	*na*	*na*	*na*		
	0.052 (0.008)	0.053 (0.009)	*na*	*na*	*na*		
*Ostreopsis rhodesiae*	0.275 (0.038)	0.618 (0.034)	0.377 (0.036)	0.236 (0.036)	0.356 (0.038)	*na*	
	0.242 (0.024)	0.355 (0.024)	0.35 (0.02)	*na*	*na*	0.004 (0.003)	
	0.032 (0.007)	0.049 (0.008)	0.06 (0.008)	*na*	0.056 (0.008)	0.003 (0.002)	
*Ostreopsis* sp. 9/*O.* cf. *siamensis*	0.265 (0.029)	0.597 (0.029)	0.325 (0.03)	0.204 (0.025)	0.33 (0.031)	0.173 (0.036)	*na*
	0.253 (0.022)	0.361 (0.021)	0.342 (0.024)	*na*	*na*	0.136 (0.025)	0.003 (0.001)
	0.036 (0.007)	0.046 (0.007)	0.057 (0.008)	*na*	0.05 (0.008)	0.029 (0.007)	0.000 (0.000)

Standard error estimate(s) are shown in brackets and were obtained by a bootstrap procedure (1000 replicates).

*na* represents no data.

### Toxin analyses

LC–MS/MS analysis of oxidized cellular extracts from *Ostreopsis* strains did not detect any PLTX-like compounds above the limit of detection (LOD = 0.01 pg/cell) (Table 1). Analysis of gambierones produced by the *Coolia* strains revealed that four out of the five *Coolia tropicalis* strains had similar 44-MG cell quotas (Table 2), whereas no 44-MG was detected from one strain and the single strain of *C. malayensis*. *C. tropicalis* strains that yielded quantifiable amounts of 44-MG were also screened for gambierone, but no gambierone detected in any of those strains (LOD = 0.05 pg/cell) (Table 2).

## Discussion

### Morphological comparisons among *Ostreopsis* and *Coolia* species

*Ostreopsis* morpho-species identification is often difficult, as many species show significant intra-specific morphological plasticity and a lack of striking species-specific characters – suggesting they are cryptic or semi-cryptic species^3,18^. A given species can also encompass a wide genetic variability that is often linked to biogeography (cryptic intra-specific diversity recognized as ribotypes)^18,35–37,53^. The new species *Ostreopsis tairoto* sp. nov. belongs to a clade including the morphologically characterized taxa *O.* cf. *ovata*, *O.* cf. *siamensis* (sp. 9), and *O. rhodesiae*, as well as “ribotypes sp. 1, sp. 2, sp. 7, and sp. 8”. The closest sister lineage was *O. fattorussoi* (Figs. 14 A-C). *Ostreopsis mascarenensis* in the most basal position of this *Ostreopsis* subclade seems to be related as well. These species share several thecal plate characters (Table 3).

A curved suture between plates 1′ and 3′ was described as characteristic distinguishing feature of *O. fattorussoi*^11^. This suture can be curved also in *O.* cf. *ovata*^54–56^ and *Ostreopsis tairoto* (Figs. 2A, 3A). The opinion that “the curved suture … makes plates 1′ and 3′ approximately hexagonal while in the other *Ostreopsis* species they are pentagonal…” in Accoroni et al.^11^ is misleading. Counting sutures they are hexagonal also in other species (Table 3), but as the very short suture with the Po plate is often not recognized in lower magnification, it looks pentagonal on most images. Furthermore, the relative position of plate 1′ on the left epitheca half and the shape of plate 6′′ is not unique (this study; Verma et al.^12^). *Ostreopsis rhodesiae* cells can be of the same size as *Ostreopsis tairoto* and *O. fattorussoi* cells and it will be nearly impossible to identify such a cell by its morphology. The Po lengths can also overlap but the relative lengths of the 2′ plates might distinguish *Ostreopsis tairoto* from *O. fattorussoi* and *O. rhodesiae* (Table 3). Plate 2′′′′ was nearly symmetrical in *Ostreopsis tairoto* but few cells with asymmetrical 2′′′′ plate were observed in culture so that this possible distinguishing character is not reliable. Whether the plate is rather narrow or wide is relative for all three species and variable to a certain degree. The thecal pore sizes could be a species-specific character, but an intense study would be necessary to elaborate this, and it isn’t a character suitable for use in light microscopic identification. In conclusion, an unambiguous identification of these species is only possible by molecular methods. DNA sequencing has been used to distinguish and describe several protistan species with few or variable morphological features, in order to characterize protist lineages that principally lack morphological characteristics, and to identify and study specific protistan taxa in complex natural assemblages^57–60^. The identification of marine microalgae, including HAB species, has mostly relied on microscopic observations in the past. However, methods based on morphological characteristics have limited resolutions^61,62^. First, species identification based on traditional microscopic methods is restricted to organisms with well-documented morphological characteristics. Furthermore, these methods may misidentify organisms that are fragile to be fixed for preservation^63^. Also, cryptic species cannot be accurately distinguished during routine light microscopic observations^12,60,64^. In addition, taxonomic identification is extremely time-consuming and requires well-trained specialists. Therefore, morphological analyses alone cannot provide a complete description of microalgal diversity^57,59,65,66^. However, there is yet no taxonomic consensus about describing new species without showing diagnostic (distinctive) phenotypic traits. Hoppenrath^67^ discussed both the technical and conceptual the problems of one species concept for dinoflagellates. As discussed for protists in general by Boenigk et al.^68^, also for dinoflagellates, a consensus approach for species definitions may emerge within groups, but one approach is unlikely to encompass the whole dinoflagellate lineage.

Investigating the two species *O.* cf. *siamensis* and *O.* cf. *ovata*, David et al.^69^ found that the main differential feature between them was the presence of thecal pores of two size classes in the former and only one in the latter. A great variety of cell sizes and shapes were observed for both species^69^. Cells of *O.* cf. *siamensis* were reported to be more flattened than cells of *O.* cf. *ovata*^36,70,71^. *Ostreopsis tairoto* cells seem to overlap in size with *O.* cf. *ovata* *O.* cf. *siamensis* populations (Table 3). A distinguishing feature between *Ostreopsis tairoto* and *O.* cf. *ovata* could be the small pores documented for the new species (present study). Whether *O.* cf. *ovata* really has only one size class of pores is uncertain as Aligizaki and Nikolaidis^70^ documented a very wide range of thecal pore sizes (0.07–0.32 µm) and labelled a minute pore on their Fig. 4F. The ribotype “*Ostreopsis* sp. 1” was described as *O.* cf. *ovata* from Korea^72^. Cells were in the width range of *Ostreopsis tairoto* but less deep. The Po plate was shorter (4.8–6.8 µm) and plate 2′ only about 1.25 times the Po length (Kang et al.^72^; Fig. 3C). *Ostreopsis mascarenensis* is morphologically distinct by its large size (Table 3) and the conspicuously flattened to slightly concave epitheca^16^.

*Ostreopsis lenticularis* was originally described as relatively large species without cingulum or cell undulation and with smooth thecal plates possessing pores of two size classes^13^. Chomérat et al.^18^ re-investigated *O. lenticularis* at the type locality and provided an unambiguous description (but no formal epitypification), so that the species now can be clearly identified. The problems related to past identifications and probable synonymy, were discussed in detail by Chomérat et al.^18^. The morphological features observed in this study were generally in agreement with the species description^13,18^, except of the recognition of a third size class of pores. These medium sized pores were not visible on published scanning electron micrographs^18^. But their numbers were few and they were irregularly scattered so that it is possible that they were identified as the smallest pores of the large size class in previous studies. Oblong or kidney-shaped large pores were described for strains isolated from the South China Sea in Zhang et al.^54^ and interpreted as possible culturing plasticity by Chomérat et al.^18^.

For *Coolia malayensis* the nucleus was originally described to be located in the hyposome^31^. Its position was interpreted to be in the episome in the present study (Fig. 9F). The third apical plate (3′) was pentagonal (Fig. 10A,B,L) with wide contact (suture) to plate 5′′. It was described as quadrangular without contacting plate 5′′ by Leaw et al.^31^, but that was not clearly shown in the original SEMs, only in the drawing. Thecal pores of slightly smaller size were also visible in Fig. 4C in Leaw et al.^31^. *Coolia tropicalis* was re-described by Mohammad-Noor et al.^30^. The Po plate was shorter in the morphologically investigated strain from Rarotonga (about 5.7–7.3 µm compared to 7.2–12.0 µm for cells from Malaysia, Indonesia, Belize, and Australia; Mohammad-Noor et al.^30^). Plate 2′′′′ was in contact with 1′′′′ and 2′′′ in agreement with the original description^20^, the re-description^30^ and further reports (e.g. Momigliano et al.^73^; Nguyen^74^), but not as shown in Hoppenrath et al.^3^, Fig. 31.

### Phylogenetics of *Ostreopsis* and *Coolia* species

Results from microscopic observations and phylogenetic analyses confirm the presence of two *Ostreopsis* species in the samples studied from Rarotonga, Cook Islands and Niue. Our molecular phylogenies show a similar topology for ITS/5.8S, D1–D3 and D8–D10 of the LSU rDNA regions, albeit with different support. The phylogenetic analyses revealed the discovery of *O. tairoto*, previously an undescribed clade referred to as “*Ostreopsis* sp. 3” by Sato et al.^35^ and subsequent authors, that diverged from other clades with full nodal support. Isolate CAWD184 was originally reported in Sato et al.^35^ and was labelled “*Ostreopsis* sp. 3” with the report of sequences from the ITS/5.8S and D8-D10 LSU rDNA regions. Major sampling trips were carried out around the lagoons of Rarotonga (Fig. 1) in November 2014 and June 2017, during which several *Ostreopsis* strains were isolated from the green macroalga *Halimeda* sp. Some of these strains were genetically identical and belong to the unique “*Ostreopsis* sp. 3” ribotype. This species also appears to be closely related to “*Ostreopsis* sp. 8”, *O. mascarenensis*, “*O*. sp. 4”, *O*. *fattorussoi, O. rhodesiae* and *O.* cf. *siamensis*. So far, this species has only been reported in the South Pacific, namely from Cook Islands, Niue, and Kermadecs Islands, suggesting that the species is more suitable to warm tropical waters. A similar trend has also been suggested for other *Ostreopsis* species such as *O. siamensis*^19^ (previously known as “*Ostreopsis* sp. 6”), and *O. lenticularis* (Chomérat et al.^18^) which was also isolated in this study.

Considerable levels of intra-specific variations amongst *O. tairoto* strains in the ITS/5.8S and LSU rDNA regions were observed, with strains isolated from the same island location clustering together (Figs. 14A–C). Similar levels of intra-specific variation in ribosomal genes have also been reported in other *Ostreopsis* species, such as *Ostreopsis siamensis* and *O.* cf. *ovata*, and may suggest the existence of cryptic/ pseudo-cryptic species amidst these clades^19^. Low levels of intra-specific variation have also been observed in LSU rDNA region in *O.* cf*. siamensis, O. lenticularis* and *O. rhodesiae*^12,18,75^*.* Sato et al.^35^ suggested that the evolutionary divergence within the ITS region in an *Ostreopsis* species could be reflective of wider genomic heterogeneity, indicating a wide adaptive potential to changing environments. If this were true, it would suggest that *O. tairoto* may be more genetically diverse and have a greater adaptive potential than its more genetically homogeneous counterparts. It has been suggested that genetic *p* distances of > 0.040 in the ITS rDNA region could be used to delineate dinoflagellate species^76^. While intra-specific genetic diversity in this study was found to be 0.048 (Table 4), all subclades cluster together in a monophyletic clade, therefore, we see presently no reason for subdividing *O. tairoto*. Also, such differentiation would not be complete without the identification of compensatory base changes in the ITS2 secondary structure, as demonstrated with the *Alexandrium ostenfeldii* complex^77^. However, due to the high genetic variability in the ITS region of *O. tairoto*, we cannot exclude that future studies may reveal that *O. tairoto* should be divided into several cryptic/ pseudo-cryptic species.

In addition, *Coolia malayensis* and *C. tropicalis* were also isolated with *Ostreopsis* species in this study. Both species are known to occur worldwide in tropical waters and have been reported from Rarotonga lagoons previously^22^. The phylogenetic trees based on partial D1-D3 LSU rDNA and ITS/5.8S rDNA sequences yielded the same branching patterns and main clades previously reported in the literature, with *C. monotis*, *C. malayensis* and *C. santacroce* as closely related species, and *C. palmyrensis* emerging as a basal lineage to those species^21,30,31,33,34^. The *C. canariensis* complex yielded three and four well-delimited phylotypes based on ITS/5.8S and D1-D3 rDNA analyses respectively, following Nascimento et al.^78^ and Phua et al.^79^, and were closely related to the newly described *C. guanchica*^34^*. C. tropicalis* emerged as the basal clade in both analyses (Fig. 15A,B).

### Toxin analyses

Strains of *Ostreopsis tairoto* and *O. lenticularis* were not identified to produce any known PLTX -like compounds based on the LC–MS/MS analysis used in this study^80^ (Table 1). Chomérat et al.^18^ screened 19 strains of *O. lenticularis* from French Polynesia and none of the tested strains showed toxic activity on neuroblastoma cells, while LC–MS/MS analyses performed on the strains from Tahiti Island (i.e. the type locality) confirmed that PLTX and related structural analogues were below the LOD. All strains of *Ostreopsis tairoto* that have been screened to date have not produced PLTX-like compounds above the LOD of the LC–MS/MS method^52,80^. Numerous PLTX-like compounds (such as isobaric PLTX, ovatoxins-a–k, ostreocin-A, -B, -D, -E1 and mascarenotoxin-A–C) have been described from several *Ostreopsis* species to date^45,81–84^. However, the LC/MS–MS approach used in our study monitors sub-structures generated by the oxidative cleavage of large PLTX-like compounds and cannot differentiate between many of the toxin analogues. However, all PLTX-like compounds have a common sub-structure that is monitored during the analysis. The absence of this sub-structure in the *Ostreopsis* isolates tested during this study supports the statement that these strains do not produce any PLTX-like compounds.

44-MG was detected in three out of the five *C. tropicalis* strains analysed in this study. However, the *C. malayensis* strain tested in this study did not produce 44-MG. 44-MG has been detected from several *Gambierdiscus* species/strains, namely *G. australes*, *G. belizeanus*, *G. caribaeus*, *G. carpenteri*, *G. cheloniae*, *G. holmesii*, *G. honu*, *G. lapillus*, *G. lewisii*, *G. pacificus* and *G. polynesiensis*^85^. The compound has also been detected in *Fukuyoa* species, C*. malayensis* and *C. tropicalis*^85^. Toxicity within the genus *Coolia* was first reported in the early 1980s, based on hemolytic activity via in vitro assays, although no toxicity to mice and fish was registered for the same methanol extract^86^. Since then, this genus of benthic dinoflagellates has been considered potentially toxic^3^. According to Boente-Juncal et al.^87^, 44-MG exhibits similar biological activities to gambierone and CTX-3B, although at a much lower rate, leading to the decreased viability of undifferentiated neuroblastoma (N2a) cells and modified expression of excitatory neurotransmitter receptor subunits. However, in contrast, the study by Murray et al.^85^ demonstrated 44-MG had an LD_50_ by intraperitoneal injection to mice of 20–38 mg/kg, which is essential non-toxic. This finding has been supported by a recent study that Stuart et al.^88^ conducted, where 44-MG was assessed using the N2a cell-based assay and only displayed a minor toxic effect when dosed at the highest concentration trialled (4.8 µg/mL).

## Materials and methods

### Sample collection, strain establishment and growth

Samples were collected from several sites at Rarotonga, Cook Islands and Niue between November 2014 and July 2019 during research expeditions as described in Smith et al.^89^ (Fig. 1 and Tables 1,2). Macroalgal substrates were collected in sealable plastic containers with local sea water (approx. 500 mL each) from which epiphytic dinoflagellate cells were detached by vigorous shaking. Germanium dioxide (approx. 1% final conc.) (Sigma-Aldrich) was added to the suspended phytoplankton community to suppress diatom growth. Single *Ostreopsis* and *Coolia* cells were isolated under an inverted light microscope and transferred to a drop of clean filtered seawater. The transfer was repeated until no nano- and pico-planktonic cells were observed in the cell’s vicinity under the microscope. Monoclonal isolates of *Ostreopsis* and *Coolia* were established in 5 × diluted f/2 medium^90^ in a 24–multi well culture plate (Corning Life Sciences, Durham, USA) with 1 mL medium, and transported back to the laboratories at University of Technology Sydney, Australia and Cawthron Institute, New Zealand. On return to the labs, cultures were grown at 25 °C at a salinity of 35, with 40–100 μmol photons m^−2^ s^−1^ irradiance (12:12 h L:D). Cultures were maintained in the same conditions thereafter and subcultured once every 3–4 weeks.

### Microscopy

Living and fixed cells were picked under a Leica DMIL inverted microscope (Leica Microsystems GmbH, Wetzlar, Germany), placed on an object slide and observed with a Leica DMRB (Leica Microsystems GmbH, Wetzlar, Germany) equipped with differential interference contrast optics at 400 × and 640 × magnification with oil immersion objectives. Digital photos were taken using a Leica DFC420C camera (Leica Microsystems GmbH, Wetzlar, Germany). Fixed cells of the new *Ostreopsis* species were stained with Solophenyl Flavine 7GFE 500 (Ciba Specialty Chemicals, High Point, North Carolina, USA) as described by Chomérat et al.^91^ and thecal plates visualized by epifluorescence microscopy. Cell dimensions were measured under 400 × using a calibrated eyepiece of Eclipse TS100 inverted microscope with bright field optics (Nikon, Hilton, Australia). Cells were harvested from the culture medium via centrifugation and fixed in 1% Lugol solution (Sigma-Aldrich) to measure the depth = dorso-ventral diameter (DV) and transdiameter width (W) using ImageJ v1.48^92^.

For SEM, the cultures and field samples were fixed in 1% Lugol solution and stored in the dark. Cells were placed on a 5 µm Millipore filter, rinsed in distilled water, and dehydrated in a series of increasing ethanol concentrations (30, 50, 70, 85, 95, 100%), followed by chemical drying with hexamethyldisilazane at room temperature for 20 min and finally at 50 °C in a drying oven for 5 min. The sample/filter was mounted on a stub and sputter coated with gold–palladium (Bal-Tec SCD 050; BAL-TEC Präparationsgerätevertrieb, Wallof, Germany). Cells were observed using a Tescan VEGA3 microscope (Elekronen-Optik-Service GmbH, Dortmund, Germany) at 15 kV using the SE detector.

### DNA extraction, PCR amplification and phylogenetic analyses

Genomic DNA from cultures held at Cawthron Institute, New Zealand was extracted according to the protocols described in Smith et al.^89^, i.e. dinoflagellate cultures were centrifuged (50 mL; 542*g*; 10 min; room temperature) and DNA extracted using PowerSoil DNA isolation kit (Qiagen) as per manufacturer’s instructions. DNA from cultures maintained at UTS was extracted using modified 3% Cetyltrimethyl ammonium bromide (CTAB) buffer as described in Verma et al.^12^. Briefly, cell pellets were harvested via centrifugation (15 mL; 2300*g*; 10 min; room temperature) and lysed using 500 µL of buffer on a heat block at 68 °C for 2 h. The aqueous layer was separated using 24:1 chloroform: isoamyl alcohol (Sigma-Aldrich) and precipitated in 2-propanol (Sigma-Aldrich) and 3 M sodium acetate (pH 5.2) (Sigma-Aldrich). The DNA pellet was washed with 70% ethanol (Sigma-Aldrich) and vacuum dried to remove any traces of ethanol. Sterile Milli-Q water was added to DNA pellets and were stored at 20 °C until further analysis.

The partial regions of LSU rRNA gene (D1-D3 and D8-D10 regions) and the internal transcribed spacer regions and 5.8S rRNA gene (ITS/5.8S) were amplified and sequenced using primers and protocols described in Verma et al.^93^ (Supplementary Table 1). Briefly, all PCR reactions were performed in 25 μL reaction volumes containing 12.5 μL of 2 × Immomix (Bioline, Sydney, Australia), 7.5 pmol of each primer, 1 μg/μL of BSA (Biolabs, Arundel, Australia), 1 μL of template DNA and PCR grade water to give the final volume. Thermocycling conditions consisted of an initial denaturing step of 95 °C for 10 min, followed by 35 cycles of 95 °C for 20 s, 30 s annealing (Supplementary Table 1), 72 °C for 1 min and a final extension of 72 °C for 7 min. PCR products were purified with DNA Clean and Concentrator (Zymo Research, Irvine, USA) according to the manufacturer’s protocol and sequenced using a commercial service (Macrogen Inc., Seoul, Korea).

Analyses on all regions of rDNA were conducted separately. The forward and reverse sequences were trimmed, aligned, and visually refined using Geneious Prime v2020.0.5^94^. The obtained sequences were aligned with reference sequences retrieved from the National Center for Biotechnology Information (NCBI) GenBank database (http://www.ncbi.nlm.nih.gov). Multiple sequence alignments were performed using ClustalW v1.6 program as implemented in MEGA v7^95^. Substitution models were selected for each dataset based on lowest Bayesian information criterion (BIC) as a measure of the relative quality of the models using MODELTEST^96^. Phylogenetic analysis was performed using both a maximum likelihood (ML) and Bayesian inference (BI) approach. ML trees were produced in MEGA v7 using general time reversible (GTR) + gamma (G) + invariant sites (I) with 5 gamma categories substitution model for all sequence analyses. Nodal support of the ML tree was estimated via bootstrap algorithm with 1,000 replications. Bayesian analysis was performed using MrBayes v3.2.2^97^ as implemented in Geneious Prime v2020.0.5 using general time reversible model (GTR) + G model for all analyses. Four independent Markov Chain Monte Carlo (MCMC) simulations were run simultaneously for 2 × 10^6^ generations. Trees were sampled every 1 × 10^3^ generations and 1 × 10^3^ trees were discarded as burn- in. Genetic distance (pairwise uncorrected *p* distance) was estimated from the ITS/5.8S, D1-D3 and D8-D10 LSU rDNA sequences using the *p* distance model and bootstrap procedure (1 × 10^3^ replicates) in MEGA v7. All positions containing gaps and missing data were eliminated for the analyses.

### Toxin analyses

*Ostreopsis* and *Coolia* cell pellets were harvested in late stationary phase via centrifugation (50 mL; 2300*g*; 10 min; room temperature) to obtain a cell pellet of approx. 1 × 10^6^ cells. The growth medium was decanted, and the resulting pellets were frozen at − 20 °C until further analysis. Toxin extractions were completed at the Cawthron Institute using methods described in Selwood et al.^80^ for screening of PLTX-like compounds, and Murray et al.^98^ for screening of gambierone and 44-MG respectively.

Briefly, Selwood et al.^80^ monitors substructures of PLTX-like compounds that are generated via oxidative cleavage, using periodic acid, of vicinal diol groups present in intact toxin molecules. This yields an amino-aldehyde common to known PLTX-like compounds, used for quantification, and an amide-aldehyde that varies depending on the toxin analogue. A commercially available PLTX standard was used to generate a calibration curve and enable unambiguous identification of the oxidation products. The limit of detection (LOD) was determined as 0.5 ng/ml for the PLTX amine fragment, which equates to 0.01 pg/cell in an extract. The relative standard deviation of repeatability for LC–MS of oxidized PLTX standards was < 10% and < 8% for amino aldehyde and amide aldehyde, respectively, at 1 or 2 ng/ml, making this method suitable for monitoring trace levels of PLTX-like compounds.

For detection and quantification of gambierones, each *Coolia* cell pellet was extracted twice with 90% aqueous methanol, at a ratio of 1 mL per 2 × 10^5^ cells, and subsequently lysed via ultrasonication (10 min at 59 kHz). Cellular debris was removed by centrifugations (3200*g*; 5 min; 4 °C) and the supernatant transferred to another vial. The resulting supernatants were pooled to give a final extract concentration equivalent to 1 × 10^5^ cells/mL. The combined extracts were stored at − 20 °C for 24–48 h to precipitate insoluble matrix co-extractives, which were removed using centrifugation (3200*g*; 5 min; 4 °C) prior to analysis. An aliquot of the clarified extract was transferred into a 2 mL glass autosampler vial and analysed using a modification of the LC–MS/MS method described in Murray et al.^99^. The compounds are identified based on retention time (2.54 and 2.58 min for gambierone and 44-MG respectively) and fragment ion ratios compared to purified reference material, on a Waters Xevo TQ-S triple quadrupole mass spectrometer coupled to a Waters Acquity UPLC i-Class with a flowthrough needle sample manager. Data acquisition and processing was performed using MassLynx and TargetLynx software (Waters), respectively. Quantitation was achieved using a five-point, for gambierone and 44-MG (1–1000 ng/mL), linear regression calibration prepared in 90% aqueous methanol. The limit of quantification (LOQ) was determined as 0.05 pg/cell in an extract generated from a cell pellet of 1 × 10^6^ cells.

## Supplementary Information

Supplementary Information.

Supplementary Figure 1.

## Data Availability

The authors declare that all data is available on request to the corresponding author.

## References

[1] Verma A (2019). The genetic basis of toxin biosynthesis in dinofagellates. Microorganisms.

[2] Hallegraeff GM (2010). Ocean climate change, phytoplankton community responses, and harmful algal blooms: A formidable predictive challenge1. J. Phycol..

[3] Hoppenrath, M., Murray, S., Chomérat, N., Horiguchi, T. Marine Benthic Dinoflagellates - Unveiling Their Worldwide Biodiversity (Kleine Senckenberg-reihe 54). E. Schweizerbart’sche Verlagbuchhandlung (2014).

[4] Luo Z (2017). Cryptic diversity within the harmful dinoflagellate *Akashiwo sanguinea* in coastal Chinese waters is related to differentiated ecological niches. Harmful Algae.

[5] Litaker RW (2009). Taxonomy of *Gambierdiscus* including four new species, *Gambierdiscus caribaeus*, *Gambierdiscus carolinianus*, *Gambierdiscus carpenteri* and *Gambierdiscus ruetzleri* (Gonyaulacales, Dinophyceae). Phycologia.

[6] Hoppenrath M (2013). Taxonomy and phylogeny of the benthic *Prorocentrum* species (Dinophyceae)—A proposal and review. Harmful Algae.

[7] Wells ML (2020). Future HAB science: Directions and challenges in a changing climate. Harmful Algae.

[8] Rhodes L (2011). World-wide occurrence of the toxic dinoflagellate genus *Ostreopsis* Schmidt. Toxicon.

[9] Parsons ML (2012). *Gambierdiscus* and *Ostreopsis*: Reassessment of the state of knowledge of their taxonomy, geography, ecophysiology, and toxicology. Harmful Algae.

[10] Schmidt J (1901). Preliminary report of the botanical results of the Danish expedition to Siam (1899–1900). Part IV Peridiniales. Bot. Tidsskr..

[11] Accoroni S (2016). *Ostreopsis fattorussoi* sp. nov. (Dinophyceae), a new benthic toxic *Ostreopsis* species from the eastern Mediterranean Sea. J. Phycol..

[12] Verma A, Hoppenrath M, Dorantes-Aranda JJ, Harwood DT, Murray SA (2016). Molecular and phylogenetic characterization of *Ostreopsis* (Dinophyceae) and the description of a new species, *Ostreopsis*
*rhodesae* sp. nov., from a subtropical Australian lagoon. Harmful Algae.

[13] Fukuyo Y (1981). Taxonomical study on benthic dinoflagellates collected in coral reefs. Nippon Suisan Gakk..

[14] Faust MA (1999). Three new *Ostreopsis* species (Dinophyceae): *O*. *marinus* sp. nov., *O*. *belizeanus* sp. nov., and *O*. *caribbeanus* sp. nov.. Phycologia.

[15] Faust MA, Morton SL (1995). Morphology and ecology of the marine dinoflagellate *Ostreopsis*
*labens* sp. nov. (Dinophyceae). J. Phycol..

[16] Chomérat N, Bilien G, Couté A, Quod J-P (2020). Reinvestigation of *Ostreopsis mascarenensis* Quod (Dinophyceae, Gonyaulacales) from Reunion Island (SW Indian Ocean): Molecular phylogeny and emended description. Phycologia.

[17] Boisnoir A, Bilien G, Lemée R, Chomérat N (2022). First insights on the diversity of the genus *Ostreopsis* (Dinophyceae, Gonyaulacales) in Guadeloupe Island, with emphasis on the phylogenetic position of *O*. *heptagona*. Eur. J. Protistol..

[18] Chomérat N (2019). *Ostreopsis lenticularis* Y. Fukuyo (Dinophyceae, Gonyaulacales) from French Polynesia (South Pacific Ocean): A revisit of its morphology, molecular phylogeny and toxicity. Harmful Algae.

[19] Nguyen-Ngoc L (2021). Morphological and genetic analyses of *Ostreopsis* (Dinophyceae, Gonyaulacales, Ostreopsidaceae) species from Vietnamese waters with a re-description of the type species, *O*. *siamensis* 1. J. Phycol..

[20] Faust MA (1995). Observation of sand-dwelling toxic dinoflagellates (Dinophyceae) from widely differing sites, including two new species. J. Phycol..

[21] David H, Laza-Martínez A, Miguel I, Orive E (2014). Broad distribution of *Coolia monotis* and restricted distribution of *Coolia* cf. *canariensis* (Dinophyceae) on the Atlantic coast of the Iberian Peninsula. Phycologia.

[22] Rhodes LL (2010). Toxic dinoflagellates (Dinophyceae) from Rarotonga Cook Islands. Toxicon.

[23] Meunier A (1919). *Coolia monotis* sp. nov. in Mémoires du Musée Royal d'Histoire Naturelle de Belgique. Microplankton Mer Flamande, Méme partie—Les Péridiniens.

[24] Rhodes L (2014). Epiphytic dinoflagellates in sub-tropical New Zealand, in particular the genus *Coolia* Meunier. Harmful Algae.

[25] Rhodes L, Adamson J, Suzuki T, Briggs L, Garthwaite I (2000). Toxic marine epiphytic dinoflagellates, *Ostreopsis siamensis* and *Coolia monotis* (Dinophyceae), in New Zealand. N. Z. J. Mar. Freshw. Res..

[26] Fraga S, Penna A, Bianconi I, Paz B, Zapata M (2008). *Coolia*
*canariensis* sp. nov. (Dinophyceae), a new nontoxic epiuphytic benthic dinoflagellate from the Canary Islands 1. J. Phycol..

[27] Lindemann, E. Abteilung Peridineae (Dinoflagellate). *In* Die Natürlichen Pflanzenfamilien nebst ihren Gattungen und wichtigeren Arten insbesondere den Nutzpflanzen, 3–104 (1928).

[28] Biecheler B (1952). Recherches sur les Péridiniens. Bulletin biologique de France et de Belgique Supplement.

[29] Balech E (1956). Étude des dinoflagellés du sable de Roscoff. Revue Algologique, Nouvelle Serie.

[30] Mohammad-Noor N (2013). Autecology and phylogeny of *Coolia tropicalis* and *Coolia malayensis* (Dinophyceae), with emphasis on taxonomy of *C*. *tropicalis* based on light microscopy, scanning electron microscopy and LSU r DNA 1. J. Phycol..

[31] Leaw CP, Lim PT, Cheng KW, Ng BK, Usup G (2010). Morphology and molecular characterization of a new species of thecate benthic dinoflagellate, *Coolia*
*malayensis* sp. nov. (Dinophyceae) 1. J. Phycol..

[32] Ten-Hage L, Turquet J, Quod J, Couté A (2000). *Coolia*
*areolata* sp. nov. (Dinophyceae), a new sand-dwelling dinoflagellate from the southwestern Indian Ocean. Phycologia.

[33] Karafas S, York R, Tomas C (2015). Morphological and genetic analysis of the *Coolia monotis* species complex with the introduction of two new species, *Coolia*
*santacroce* sp. nov. and *Coolia*
*palmyrensis* sp. nov. (Dinophyceae). Harmful Algae.

[34] David H, Laza-Martínez A, Rodríguez F, Fraga S, Orive E (2020). *Coolia*
*guanchica* sp. nov.(Dinophyceae) a new epibenthic dinoflagellate from the Canary Islands (NE Atlantic Ocean). Eur. J. Phycol..

[35] Sato S (2011). Phylogeography of *Ostreopsis* along west Pacific coast, with special reference to a novel clade from Japan. PLoS One.

[36] Penna A (2005). Characterization of *Ostreopsis* and *Coolia* (Dinophyceae) isolates in the western Mediterranean Sea based on morphology, toxicity and internal transcribed spacer 5.8 S rDNA sequences. J. Phycol..

[37] Tawong W (2014). Distribution and molecular phylogeny of the dinoflagellate genus *Ostreopsis* in Thailand. Harmful Algae.

[38] Faimali M (2012). Toxic effects of harmful benthic dinoflagellate *Ostreopsis ovata* on invertebrate and vertebrate marine organisms. Mar. Environ. Res..

[39] Tubaro A (2011). Case definitions for human poisonings postulated to palytoxins exposure. Toxicon.

[40] Ciminiello P (2006). Investigation of the toxin profile of Greek mussels *Mytilus galloprovincialis* by liquid chromatography mass spectrometry. Toxicon.

[41] Giussani V (2015). Active role of the mucilage in the toxicity mechanism of the harmful benthic dinoflagellate *Ostreopsis* cf. *ovata*. Harmful Algae.

[42] Usami M (1995). Palytoxin analogs from the dinoflagellate *Ostreopsis siamensis*. J. Am. Chem. Soc..

[43] Ukena T (2001). Structure elucidation of ostreocin D, a palytoxin analog isolated from the dinoflagellate *Ostreopsis siamensis*. Biosci. Biotechnol. Biochem..

[44] Amzil Z (2012). Ovatoxin-a and palytoxin accumulation in seafood in relation to *Ostreopsis* cf. *ovata* blooms on the French Mediterranean coast. Mar. Drugs.

[45] Ciminiello P (2012). Unique toxin profile of a Mediterranean *Ostreopsis* cf. *ovata* strain: HR LC-MS n characterization of ovatoxin-f, a new palytoxin congener. Chem. Res. Toxicol..

[46] Laza-Martinez A, Orive E, Miguel I (2011). Morphological and genetic characterization of benthic dinoflagellates of the genera *Coolia*, *Ostreopsis* and *Prorocentrum* from the south-eastern Bay of Biscay. Eur. J. Phycol..

[47] Holmes MJ, Lewis RJ, Jones A, Hoy AWW (1995). Cooliatoxin, the first toxin from *Coolia monotis* (Dinophyceae). Nat. Toxins.

[48] Rhodes LL, Thomas AE (1997). *Coolia monotis* (Dinophyceae): A toxic epiphytic microalgal species found in New Zealand (Note). N. Z. J. Mar. Freshw. Res..

[49] Tibiriçá CEJdA (2020). Diversity and toxicity of the genus *Coolia* Meunier in Brazil, and detection of 44-methyl Gambierone in *Coolia tropicalis*. Toxins.

[50] Tillmann U, Hoppenrath M, Gottschling M (2019). Reliable determination of *Prorocentrum micans* Ehrenb. (Prorocentrales, Dinophyceae) based on newly collected material from the type locality. Eur. J. Phycol.

[51] Chomérat N (2020). Taxonomy and toxicity of a bloom-forming *Ostreopsis* species (Dinophyceae, Gonyaulacales) in Tahiti island (South Pacific Ocean): One step further towards resolving the identity of *O*. *siamensis*. Harmful Algae.

[52] Rhodes LL (2017). The dinoflagellate genera *Gambierdiscus* and *Ostreopsis* from subtropical Raoul Island and North Meyer Island, Kermadec Islands. N. Z. J. Mar. Freshw. Res..

[53] Penna A (2010). A phylogeographical study of the toxic benthic dinoflagellate genus *Ostreopsis* Schmidt. J. Biogeogr..

[54] Zhang H (2018). Morphology and molecular phylogeny of *Ostreopsis* cf. *ovata* and *O*. *lenticularis* (Dinophyceae) from Hainan Island South China Sea. Phycol. Res..

[55] Carnicer O, García-Altares M, Andree KB, Diogène J, Fernández-Tejedor M (2016). First evidence of *Ostreopsis* cf. *ovata* in the eastern tropical Pacific Ocean Ecuadorian coast. Bot. Mar..

[56] Nascimento SM (2020). *Ostreopsis* cf. *ovata* (Dinophyceae) molecular phylogeny, morphology, and detection of ovatoxins in strains and field samples from Brazil. Toxins.

[57] Caron DA (2009). Defining DNA-based operational taxonomic units for microbial-eukaryote ecology. Appl. Environ. Microbiol..

[58] McManus GB, Katz LA (2009). Molecular and morphological methods for identifying plankton: What makes a successful marriage?. J. Plankton Res..

[59] De Vargas C (2015). Eukaryotic plankton diversity in the sunlit ocean. Science.

[60] del Campo J (2016). Ecological and evolutionary significance of novel protist lineages. Eur. J. Protistol..

[61] Hallegraeff G (2003). Harmful algal blooms: A global overview. Man. Harmful Mar. Microalgae.

[62] Penna A, Casabianca S, Guerra AF, Vernesi C, Scardi M (2017). Analysis of phytoplankton assemblage structure in the Mediterranean Sea based on high-throughput sequencing of partial 18S rRNA sequences. Mar. Genom..

[63] Zarauz L, Irigoien X (2008). Effects of Lugol’s fixation on the size structure of natural nano–microplankton samples, analyzed by means of an automatic counting method. J. Plankton Res..

[64] De Luca D, Piredda R, Sarno D, Kooistra WH (2021). Resolving cryptic species complexes in marine protists: phylogenetic haplotype networks meet global DNA metabarcoding datasets. ISME J..

[65] Wang Z (2022). Phytoplankton community and HAB species in the South China Sea detected by morphological and metabarcoding approaches. Harmful Algae.

[66] Le Bescot N (2016). Global patterns of pelagic dinoflagellate diversity across protist size classes unveiled by metabarcoding. Environ. Microbiol..

[67] Hoppenrath M (2017). Dinoflagellate taxonomy—A review and proposal of a revised classification. Mar. Biodivers..

[68] Boenigk J, Ereshefsky M, Hoef-Emden K, Mallet J, Bass D (2012). Concepts in protistology: Species definitions and boundaries. Eur. J. Protistol..

[69] David H, Laza-Martínez A, Miguel I, Orive E (2013). *Ostreopsis* cf. *siamensis* and *Ostreopsis* cf. *ovata* from the Atlantic Iberian Peninsula: Morphological and phylogenetic characterization. Harmful Algae.

[70] Aligizaki K, Nikolaidis G (2006). The presence of the potentially toxic genera *Ostreopsis* and *Coolia* (Dinophyceae) in the North Aegean Sea Greece. Harmful Algae.

[71] Selina MS, Orlova TY (2010). First occurrence of the genus *Ostreopsis* (Dinophyceae) in the Sea of Japan. Bot. Mar..

[72] Kang NS (2013). Morphology and molecular characterization of the epiphytic benthic dinoflagellate *Ostreopsis* cf. *ovata* in the temperate waters off Jeju Island Korea. Harmful Algae.

[73] Momigliano P, Sparrow L, Blair D, Heimann K (2013). The diversity of *Coolia* spp. (Dinophyceae Ostreopsidaceae) in the central Great Barrier Reef region. PloS One.

[74] Nguyen LN (2014). Morphology and distribution of the three epiphytic dinoflagellate species *Coolia monotis*, *C*. *tropicalis*, and *C*. *canariensis* (Ostreopsidaceae, Gonyaulacales, Dinophyceae) from Vietnamese coastal waters. Ocean Sci..

[75] Verma A (2020). Functional significance of phylogeographic structure in a toxic benthic marine microbial eukaryote over a latitudinal gradient along the East Australian Current. Ecol. Evol..

[76] Wayne Litaker R (2007). Recognizing dinoflagellate species using ITS rDNA sequences 1. J. Phycol..

[77] Kremp A (2014). Phylogenetic relationships, morphological variation, and toxin patterns in the *Alexandrium ostenfeldii* (D inophyceae) complex: Implications for species boundaries and identities. J. Phycol..

[78] Nascimento SM, da Silva RA, Oliveira F, Fraga S, Salgueiro F (2019). Morphology and molecular phylogeny of *Coolia tropicalis*, *Coolia malayensis* and a new lineage of the *Coolia canariensis* species complex (Dinophyceae) isolated from Brazil. Eur. J. Phycol..

[79] Phua YH, Roy MC, Lemer S, Husnik F, Wakeman KC (2021). Diversity and toxicity of Pacific strains of the benthic dinoflagellate *Coolia* (Dinophyceae), with a look at the *Coolia canariensis* species complex. Harmful Algae.

[80] Selwood AI (2012). A sensitive assay for palytoxins, ovatoxins and ostreocins using LC-MS/MS analysis of cleavage fragments from micro-scale oxidation. Toxicon.

[81] Ciminiello P (2012). Isolation and structure elucidation of ovatoxin-a, the major toxin produced by *Ostreopsis ovata*. J. Am. Chem. Soc..

[82] Dell’Aversano, C. et al.* Ostreopsis* cf. *ovata* from the Mediterranean area. Variability in toxinprofiles and structural elucidation of unknowns through LC-HRMSn. In Proc. of the 16th International Conference on Harmful Algae, 70–73 (2014).

[83] Terajima T, Uchida H, Abe N, Yasumoto T (2019). Structure elucidation of ostreocin-A and ostreocin-E1, novel palytoxin analogs produced by the dinoflagellate *Ostreopsis siamensis*, using LC/Q-TOF MS. Biosci. Biotechnol. Biochem..

[84] Tartaglione L (2016). Chemical, molecular, and eco-toxicological investigation of *Ostreopsis* sp. from Cyprus Island: Structural insights into four new ovatoxins by LC-HRMS/MS. Anal. Bioanal. Chem..

[85] Murray JS (2020). The role of 44-methylgambierone in ciguatera fish poisoning: Acute toxicity, production by marine microalgae and its potential as a biomarker for *Gambierdiscus* spp. Harmful Algae.

[86] Nakajima I, Oshima Y, Yasumoto T (1981). Toxicity of benthic dinoflagellates found in coral reef. Toxicity of benthic dinoflagellates in Okinawa. Nippon Suisan Gakk..

[87] Boente-Juncal A (2019). Structure elucidation and biological evaluation of maitotoxin-3, a homologue of gambierone, from *Gambierdiscus belizeanus*. Toxins.

[88] Stuart J (2022). Geographical distribution, molecular and toxin diversity of the dinoflagellate species *Gambierdiscus honu* in the Pacific region. Harmful Algae.

[89] Smith KF (2016). A new *Gambierdiscus* species (Dinophyceae) from Rarotonga, Cook Islands: *Gambierdiscus cheloniae* sp. nov. Harmful Algae.

[90] Guillard RRL (1975). Culture of Marine Invertebrates Animals.

[91] Chomérat N, iti Gatti CM, Nézan É, Chinain M (2017). Studies on the benthic genus *Sinophysis* (Dinophysales, Dinophyceae) II. *S*. *canaliculata* from Rapa Island (French Polynesia). Phycologia.

[92] Abràmoff MD, Magalhães PJ, Ram SJ (2004). Image processing with ImageJ. Biophotonics Int..

[93] Verma A (2016). Molecular phylogeny, morphology and toxigenicity of *Ostreopsis* cf. *siamensis* (Dinophyceae) from temperate south-east Australia. Phycol. Res..

[94] Kearse M (2012). Geneious basic: An integrated and extendable desktop software platform for the organization and analysis of sequence data. Bioinformatics.

[95] Kumar S, Stecher G, Tamura K (2016). MEGA7: Molecular evolutionary genetics analysis version 7.0 for bigger datasets. Mol. Biol. Evol..

[96] Posada D, Crandall KA (1998). MODELTEST: Testing the model of DNA substitution. Bioinformatics.

[97] Ronquist F, Huelsenbeck JP (2003). MrBayes 3: Bayesian phylogenetic inference under mixed models. Bioinformatics.

[98] Murray JS (2021). Acute toxicity of gambierone and quantitative analysis of gambierones produced by cohabitating benthic dinoflagellates. Toxins.

[99] Murray JS, Boundy MJ, Selwood AI, Harwood DT (2018). Development of an LC-MS/MS method to simultaneously monitor maitotoxins and selected ciguatoxins in algal cultures and P-CTX-1B in fish. Harmful Algae.

