# Supplementary material for: Ostreopsis Schmidt and Coolia Meunier (Dinophyceae, Gonyaulacales) from Cook Islands and Niue (South Pacific Ocean), including description of Ostreopsis tairoto sp. nov
Source: Sci Rep. 2023 Feb 22;13:3110. doi: 10.1038/s41598-023-29969-z (PMC9947023; doi:10.1038/s41598-023-29969-z)
Supplement: Supplementary file 1 — Supplementary Information. [file 41598_2023_29969_MOESM1_ESM.pdf]

Supplementary Table 1. Primers used for amplification and sequencing, and their annealing temperatures

**Primer**	**Primer sequence**	**Target region**	**Direction**	**Ta**
**D1R**[1]	5'-ACC CGC TGA ATT TAA GCA TA-3'	28S (D1-D3)	Forward	55 ºC
**D3B**[2]	5'-TCG GAG GGA ACC AGC TAC TA-3'	28S (D1-D3)	Reverse	55 ºC
**FD8**[3]	5'-GGA TTG GCT CTG AGG GTT GGG-3'	28S (D8-D10)	Forward	62 ºC
**RB**[3]	5'-GAT AGG AAG AGC CGA CAT CGA-3'	28S (D8-D10)	Reverse	62 ºC
**GLD8_421F**[4]	5'-ACA GCC AAG GGA ACG GGC TT-3'	28S (D8-D10)	Forward	62 ºC
**GLD8_677R**[4]	5'-TGT GCC GCC CCA GCC AAA CT-3'	28S (D8-D10)	Reverse	62 ºC
**ITSA**[5]	5'-GTA ACA AGG THT CCG TAG GT-3'	ITS1-5.8S-ITS2	Forward	55 ºC
**ITSB**[5]	5'-AKA TGC TTA ART TCA GCR GG-3'	ITS1-5.8S-ITS2	Reverse	55 ºC

References

1 Scholin, C. A., Herzog, M., Sogin, M. & Anderson, D. M. Identification of group-and strain-specific genetic markers for globally distributed *Alexandrium* (Dinophyceae). II. Sequence analysis of a fragment of the LSU rRNA gene. *J. Phycol.* **30**, 999-1011 (1994).

2 Nunn, G., Theisen, B., Christensen, B. & Arctander, P. Simplicity-correlated size growth of the nuclear 28S ribosomal RNA D3 expansion segment in the crustacean order Isopoda. *J. Mol. Evol.* **42**, 211-223 (1996).

3 Chinain, M., Faust, M. A. & Pauillac, S. Morphology and molecular analyses of three toxic species of *Gambierdiscus* (Dinophyceae): *G. pacificus* sp. nov., *G. australes* sp. nov., and *G. polynesiensis* sp. nov. *J. Phycol.* **35**, 1282-1296 (1999).

4 Nishimura, T. *et al.* Genetic diversity and distribution of the ciguatera-causing dinoflagellate *Gambierdiscus* spp.(Dinophyceae) in coastal areas of Japan. *PloS ONE* **8**, e60882 (2013).

5 Sato, S. *et al.* Phylogeography of *Ostreopsis* along west Pacific coast, with special reference to a novel clade from Japan. *PloS ONE* **6**, e27983 (2011).
