# Supplementary material for: Ostreopsis Schmidt and Coolia Meunier (Dinophyceae, Gonyaulacales) from Cook Islands and Niue (South Pacific Ocean), including description of Ostreopsis tairoto sp. nov
Source: Sci Rep. 2023 Feb 22;13:3110. doi: 10.1038/s41598-023-29969-z (PMC9947023; doi:10.1038/s41598-023-29969-z)
Supplement: Supplementary file 2 — Supplementary Figure 1. [file 41598_2023_29969_MOESM2_ESM.pdf]

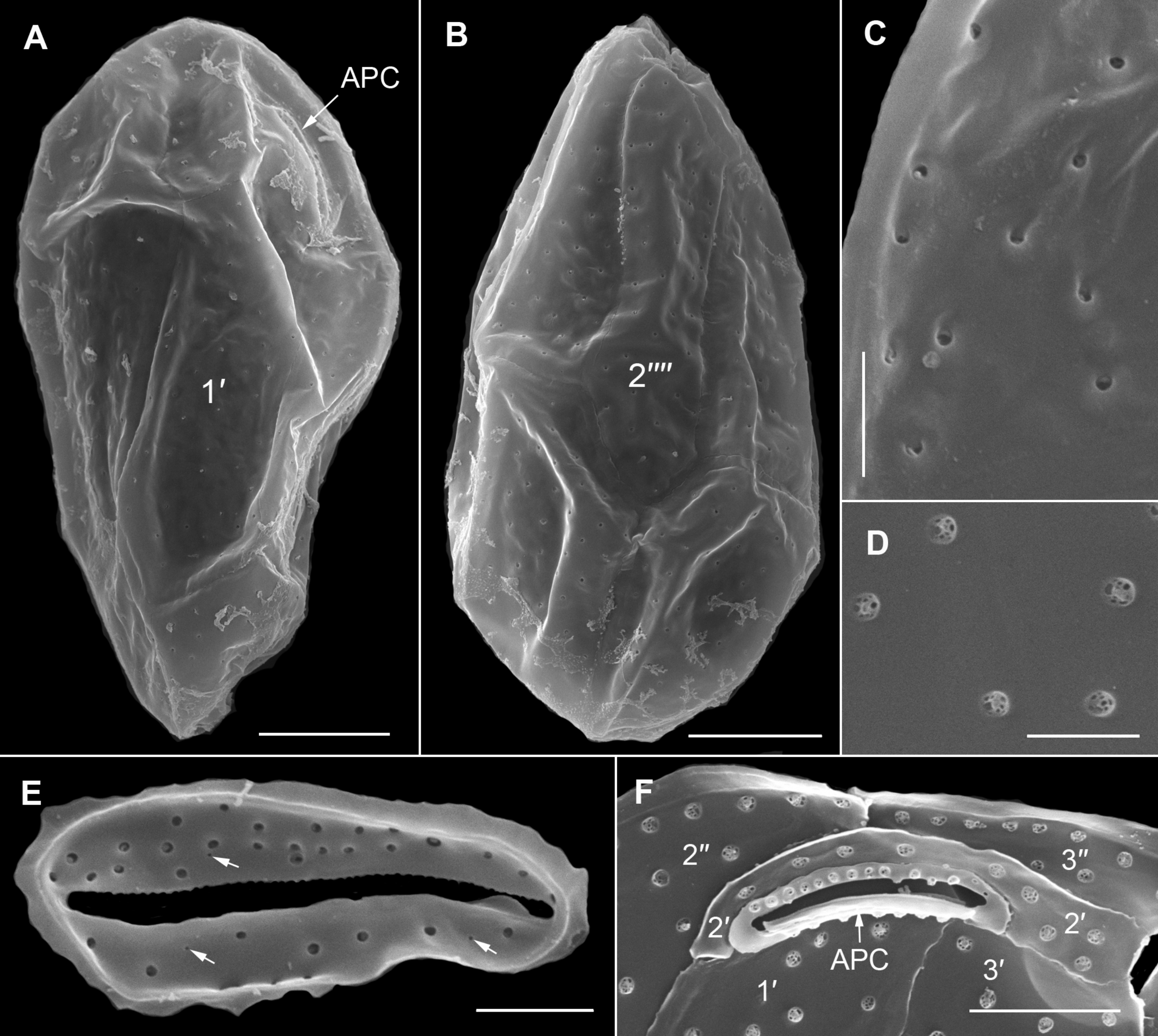
A
APC
1′
B
2′′′′
C
D
E
F
2″
3″
2′
2′
APC
1′
3′

Supplementary Fig. 1. Scanning electron micrographs of *Ostreopsis tairoto* sp. nov. strain O1C6 showing general features. A: Apical view of the epitheca. B: Antapical view of the hypotheca. C: Outside view of the main large thecal pores. D: Inside view of the main large thecal pores with inner covering perforated by irregular openings. E: Apical (outer) pore plate with slit-like apical pore. Note that there are less thecal pores in an irregular row below the apical pore and more scattered thecal pores above. F: Inside view of the left dorsal epitheca with the narrow and elongated second apical plate (2′) separating the third apical (3′) from the third precingular (3″) plate. Scale bars 10 µm in A and B, 2 µm in C-E, and 5 µm in F.
